# Trophic niche variation across the pan-Arctic coastal continuum

**DOI:** 10.1371/journal.pone.0335406

**Published:** 2025-11-04

**Authors:** Nathan D. McTigue, Katrin Iken, Ashley Ehrman, Bodil A. Bluhm, Guillaume Bridier, Rolf Gradinger, Joanna Legeżyńska, Maeve McGovern, Bailey McMeans, Frédéric Olivier, Amanda Poste, Paul E. Renaud, Virginie Roy, Janne E. Søreide, Maria Włodarska-Kowalczuk, Kenneth H. Dunton

**Affiliations:** 1 College of Fisheries and Ocean Sciences, University of Alaska Fairbanks, Fairbanks, Alaska, United States of America; 2 Marine Science Institute, The University of Texas at Austin, Port Aransas, Texas, United States of America; 3 Fisheries and Oceans Canada, Winnipeg, Manitoba, Canada; 4 Department of Arctic and Marine Biology, UiT The Arctic University of Norway, Tromsø, Norway; 5 Institut des Sciences de la Mer de Rimouski, Université du Québec À Rimouski, Rimouski, Quebec, Canada; 6 Institute of Oceanology Polish Academy of Sciences, Sopot, Poland; 7 Norwegian Institute for Water Research, Tromsø, Norway; 8 Department of Biology, University of Toronto Mississauga, Ontario, Canada; 9 Biologie des Organismes et Ecosystèmes Aquatiques, UMR MNHN/SU/UA/CNRS/IRD, Paris, France; 10 Akvaplan-niva, Tromsø, Norway; 11 Maurice Lamontagne Institute, Fisheries and Oceans Canada, Mont-Joli, Quebec, Canada; 12 Arctic Marine Biology and Ecology, University Centre in Svalbard, Longyearbyen, Norway; MARE – Marine and Environmental Sciences Centre, PORTUGAL

## Abstract

We analyzed stable carbon and nitrogen isotope values (δ^13^C and δ^15^N, respectively) for pan-Arctic coastal primary producers and consumers to detect large-scale regional trends both temporally and spatially. To facilitate comparison, we grouped coastal habitats into fjords, lagoons, shelves, and straits as four “coastscapes”. We gathered over 12,000 rows of data collected over 24 years (between 1999 and 2022) from 34 different field campaigns across the coastal Arctic (63 to 81°N and 177°W to 33°E). Our goal was to examine the isotopic patterns in pelagic and sediment particulate organic matter (pPOM and sPOM, respectively) and four consumer groups (deposit feeders, opportunists/scavengers, predators, and suspension feeders) among the four coastscapes. We found that despite the enormous spatial range of data, both pPOM and sPOM became 2.1‰ and 2.2‰ more ^13^C-depleted per decade, respectively, with parallel decreases in the δ^13^C values in consumers. The significant decrease is likely attributed to the increased contributions of ^13^C-depleted terrestrial organic matter across the Arctic coasts from freshwater inputs and coastal erosion in concert with diminishing sea ice that supports sympagic microalgae. Across all Arctic coastscapes, consumer groups exhibited overlapping isotopic composition, notably with wide δ^13^C ranges that indicated assimilation of multiple organic matter sources, including terrestrial organic matter, organic matter derived from marine phytoplankton and sea ice algae, macroalgae, and potentially benthic microalgae or degraded organic matter. This consistent pattern across coastscapes provides evidence of the trophic plasticity possessed by Arctic consumers, how coastal food webs respond to climate warming, and the signature of terrestrialization imprinted on the pan-Arctic coastal isoscape.

## Introduction

The Arctic Ocean contains only 4.3% of the world’s ocean area but hosts a disproportionate 34% of the world’s coastline [1,2]. This expanse of coastal ecosystems lining the Arctic Ocean is subjected to the impacts of warming-induced changes from both adjacent terrestrial and marine ecosystems. The Arctic is warming at a rate four times the global average [3], which is catalyzing a variety of ecosystem-level changes on land and in the ocean that ultimately impact the coastal environment. The Arctic terrestrial landscape is undergoing warming-driven increases in precipitation [4], river discharge [5], and subsurface runoff [6], widespread thawing of permafrost [7], and accelerating rates of coastal erosion [8]. Meanwhile, accelerated decreases in ice extent, duration, and thickness have manifested in the Arctic [9,10]. The resulting longer fetch of open water promotes more frequent storms [11] that generate waves that exacerbate coastal erosion, increase sediment load, and/or deliver upwelled nutrients with the potential to enhance primary production [8,12]. The combined changes occurring in coastal ecosystems directly affect human populations and are likely more pronounced than those occurring in the open waters of the Arctic Ocean [13].

The downstream effects of these alterations in terrestrial and oceanic processes directly impact coastal food webs through changes in autochthonous primary production and the delivery of allochthonous organic matter. For example, additional freshwater inputs can increase stratification that restricts nutrient exchange into the euphotic zone limiting primary production [14]. But increased freshwater discharge paired with permafrost thawing also delivers terrestrial organic matter that is assimilated by coastal consumers [15,16], inorganic nutrients that can fuel primary production where sufficient light persists [17], and advected freshwater primary producers [18]. The change in the timing of sea ice retreat will impact the abundance and species composition of sea ice algae, and will likely favor phytoplankton production [19].

As the de-icing of the Arctic continues, increases in net primary production are disproportionately higher than increases in open water area [20], implying new terrigenous sources of nutrients are fueling coastal photosynthesis [17]. Conversely, increases in atmospheric cloudiness [21] and coastal turbidity [22] in the Arctic attenuate light transmission into coastal waters, which limits primary production. The interplay between decreasing sea ice extent, more days of open water, inputs of additional nutrients, and various factors affecting photosynthetically available radiation (PAR) results in unknown outcomes for coastal primary producers in terms of community composition, timing, and net production [23]. Given the plethora of environmental changes that are impacting primary production and carbon-source diversity, examining food web structure across time and space can signify how these changes are integrated at an ecosystem level.

Stable isotopes provide a useful and proven lens to examine trophic ecology. Hinging on the tenet that stable isotope ratios of consumers are related to those of their food sources, it is possible to use these biomarkers to calculate food source contribution and trophic position at the species level [24]. Conceptual advances in stable isotope ecology have embraced ecological niche theory [25] with quantitative devices to describe the “isotopic niche” by plotting two-dimensional stable isotope space (e.g., a δ^13^C-δ^15^N biplot), describing data behavior using spatial metrics [26], and calculating spatial overlap between groups [27,28]. This concept has persisted while the analytical applications continue to evolve with Bayesian implementation [29,30] and new metrics that are independent of sample size [31]. Patterns in stable isotope distribution (isoscapes) reveal trends in baseline resource availability [32] that can be used for applications such as ecogeochemical tracking of marine animal migration patterns [33] or regional variation in freshwater inputs [34]. Spatial (pan-Arctic) and temporal (decadal) isoscapes have been documented across the Arctic for pelagic particulate organic matter [35]. Moreover, there is a contemporary emphasis on integrating biogeochemical processes at the pan-Arctic scale to understand the future Arctic Ocean [36], including expansion of macroalgae [37], changing paradigms of under-ice blooms [38], and Arctic Basin carbon budgets [39]. Examining consumer isoscapes at the pan-Arctic, multi-decadal scale is a logical next step to continue along this scientific trajectory.

Here, stable carbon (δ^13^C) and nitrogen isotope (δ^15^N) values for pan-Arctic coastal primary producers and consumers were employed to detect potential large-scale regional trends both temporally and spatially. Upon this framework, we also take into consideration that the Arctic coastline is typified by a variety of features, including fjords, rocky shorelines, bays, gulfs, sounds, shelves, eroding bluffs, lagoons, straits, archipelagoes, deltas, and rivers fed by meteoric water and glaciers. To facilitate comparison, a reductionist approach was applied to the continuum of coastal habitats, grouping them by commonality related to food web end-member availability, which we call “coastscape” [40]. Specifically, we ask: what are the temporal and spatial patterns in isotopic niches of consumers among Arctic coastal ecosystem habitat types (coastscapes) and geographic regions? We test the following null hypotheses: (1) stable isotope values of end-members and consumers do not change on a multi-decadal scale, (2) isotopic niches of consumers exhibiting the same feeding habit do not differ among pan-Arctic coastscapes, and (3) isotopic niches of consumers from a common coastscape do not differ regionally across the Arctic. By addressing these hypotheses, we can evaluate the relative importance of different organic matter sources to consumers and assess if the delivery of terrestrial organic matter sources to coastal food webs across the Arctic is changing across time and space. Synthesizing this information at the pan-Arctic level is a novel approach to investigate trophic niche variation and explore the ecological plasticity of consumers at the land-sea-ice interface of the coastal Arctic where multiple environmental changes are occurring in tandem.

## Methods

### Data collation

A non-exhaustive survey was conducted to assemble stable carbon (δ^13^C) and nitrogen (δ^15^N) data from coastal habitats from 63 to 81 °N and 177 °W to 33 °E. Data were gathered from 34 sampling campaigns between 1999 and 2022, including published data [15,32,41–67] and other unpublished sources (see S1 Table for full details). From this, 12,189 rows of data were aggregated that satisfied the requirement of including a collection date, latitude and longitude of collection site, collection depth, both δ^13^C and δ^15^N values, and a taxonomic identification. Julian date was determined for the dataset using the earliest date of collection as the origin (i.e., zero). Stable isotope ratios from all collated data are represented in the δ notation as parts per thousand (‰) determined by the equation δX = ((R_sample_/R_standard_) – 1) x 1000, where X is either ^13^C or ^15^N, and R is the ratio ^13^C/^12^C or ^15^N/^14^N, respectively. International standards, Pee Dee Belemnite for δ^13^C (PDB: USGS 24), and atmospheric air for δ^15^N (IAEA-N-1 and 2,) were used to determine R_standard_.

### Defining coastscapes

Following the conceptual framework devised by international efforts integrating science with Indigenous and local knowledge [40], we identified common physiographic features within the coastal Arctic ecosystem continuum that ultimately influence food source availability (Table 1). Collection sites were grouped based on their physical connection to other bodies of water, potential interaction with the coastline, allochthonous organic matter delivery through glacial or riverine inputs, and macroalgal subsidy presence. Accordingly, four “coastscapes” were identified a priori as either fjord-like, lagoon-like, shelf-like, or strait-like habitats (Table 1) to facilitate subsequent analysis at the pan-Arctic scale. For simplicity, coastscapes are referred to hereafter as fjord, lagoon, shelf, or strait.

**Table 1:** Heuristics developed to identify coastscapes in the coastal Arctic habitat continuum.

Coastscape characteristic	Fjord	Lagoon	Shelf	Strait
Enclosed by barrier islands	- -	++	- -	- -
Surrounded by land on 3 sides; outlet to larger body of water may contain a sill	++	- -	- -	- -
Surrounded by 2 bodies of land with 2 outlets to water bodies on the others	- -	-	- -	++
Relatively distant from land, > 20 km	- -	- -	+	+
Receives glacial freshwater input	+	- -	- -	-
Receives riverine (meteoric water) input	-	++	+	- -
Seasonal salinity fluctuations of < 25	- -	++	-	- -
Macroalgal food web end-member available	+	-	-	+

Coastscape characteristics are denoted as ++ (almost always with few exceptions), + (oftentimes yes), - (oftentimes no), and - - (rarely with few exceptions).

### Regional Arctic sector designation

**Fig 1:**
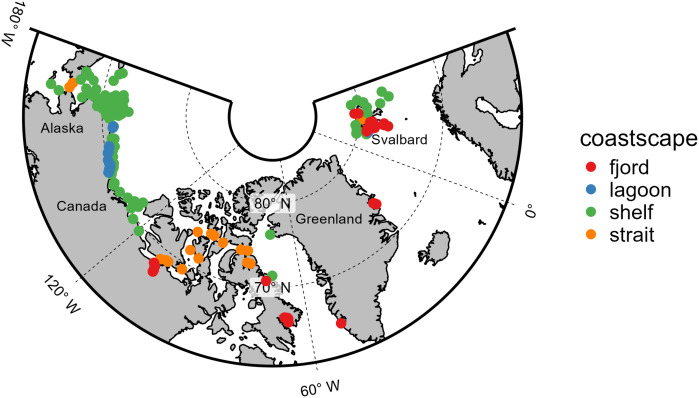
Sampling locations across the Arctic where data were aggregated. Colors denote coastscape. Each point may represent many data rows.

We are aware that the coastscape coverage in this analysis was confounded by regional distributions and spatial autocorrelation (see Fig 1). For example, fjords are absent from the Beaufort Sea and Northern Bering/Chukchi Seas regions, and straits are common in the Canadian Arctic Archipelago but only rarely represented in the rest of the study area. Therefore, in addition to the coastscapes, *a priori* regional designations were assigned to data based on collection site longitude. The Northern Bering/Chukchi Seas sector was defined as sites west of 157 °W, the Beaufort Sea sector was between 123°W and 157°W, the Canadian Arctic Archipelago sector was between 78°W and 123°W, the Baffin Bay sector was between 45°W and 78°W, the East Greenland sector was between 0° and 45°W, and the Svalbard sector was east of 0°.

### Bathymetry requirements

Sample collection depth was considered when consolidating the dataset since it has a paradoxical relationship to the distance to coastal environments in some instances. For example, depths can be > 300 m in some fjord and strait environments that are in close proximity to a coastline, whereas the broad, shallow shelves of the Chukchi and Northern Bering Seas host locations that are > 100 km from the nearest coast yet are < 100 m deep [1]. Instead of using a single bathymetric requirement across the entire dataset, collection sites deeper than 45 m in the Northern Bering, Chukchi, and Beaufort Sea shelves were omitted from analysis. Some shelf sites around Svalbard and the Canadian Archipelago were deeper than this but were retained in the dataset due to their close proximity to their respective coastlines. These restrictions distilled the dataset to 8,803 rows.

### Taxonomic classification, feeding habit assignment, and end-member synonymization

Food web end-members were collated from a 20-y period from 2 April 2002–5 August 2022 and totaled 1,078 rows. 86% of end-member data were classified as either pelagic particulate organic matter (pPOM) or sediment particulate organic matter (sPOM), while the remaining end-members consisted of sea-ice particulate organic matter (iPOM), benthic microalgae, red algae, and brown algae. Ice algae were grouped with iPOM. Generically described particulate organic matter and all phytoplankton collected from all depths were grouped as pPOM. Sediment or sediment organic matter was grouped into sPOM; when specified, sPOM was collected from the most surficial 0.5–2 cm of undisturbed surface sediment. All macroalgal species were grouped as macroalgae. Only pPOM and sPOM were tested for temporal changes since they were consistently represented throughout the collection period.

The remaining 7,725 rows of data were for consumers that consisted of 639 distinct taxa from 339 genera and 15 phyla collected between 6 May 1999 and 17 August 2022. Taxonomic classifications were attributed from the World Register of Marine Species (www.marinespecies.org). Feeding habits were assigned from either The Arctic Traits Database [68], Macdonald et al. [69], or Ehrman et al. [50]. If species-level information was not available, information from congeners or cofamilials was used. Feeding habits were aggregated into four distinct groups based on similar ecological function according to McTigue & Dunton [57]. The categorizations allowed for subsequent analysis across Arctic ecosystems at a functional level without the caveats of biogeographical species distributions limiting comparisons since few cosmopolitan species existed with sufficient replication within the dataset.

Grazers, surface deposit feeders, and subsurface deposit feeders were combined as deposit feeders. Filter feeders were combined with suspension feeders. Parasites were combined with predators. Less than 10% of data were vertebrates consisting of fish and mammals, all of which were classified into the predator feeding habit. Scavengers and omnivores were combined into the opportunist/scavengers group. The dataset was dominated by benthic taxa but included some pelagic and sympagic species. The only zooplanktonic organisms included in analysis were Copepoda, *Balanus* sp., and Pycnogonida. Bulk zooplankton and microzooplankton were not used.

### Lipid correction of stable isotope data

Lipids are generally ^13^C-depleted compared to other tissues and could potentially influence the comparison of stable carbon isotope values of organisms of varying lipid content [70]. Global mathematical corrections exist [71] but consist of broad categorizations at the level of marine fishes or marine invertebrates, for example, and the organisms used to determine the correction factors were not Arctic fauna.

A subset of aggregated data possessed stable carbon and nitrogen isotope values on paired lipid-extracted and non-extracted tissue samples from a variety of Arctic invertebrate fauna. Briefly, for select samples across a large number of invertebrate taxa and feeding types collected by the Arctic Marine Biodiversity Observation Network (AMBON) from the Chukchi Sea shelf, samples were divided into two parts. Lipids were chemically extracted from one part of each of these samples while the other remained untreated. The sub-samples selected for lipid extraction were treated at least three times in 2:1 chloroform:methanol (v:v) for at least 12 h for each extraction. After the final extraction, samples were dried and measured for stable isotope composition in addition to the non-extracted partner sample.

Comparisons among lipid-extracted and non-extracted data were made with the goal of creating taxon-specific mathematical stable isotope value corrections for lipid content. Although taxa were classified at least to the genus level within the raw data, taxa were aggregated at the phylum level to increase sample size for linear regression analysis. Wilcoxon sign rank tests (non-parametric paired t-test) were used to determine if there was a significant difference between lipid-extracted and non-extracted tissue samples. The exception to this procedure was for the copepod genus *Calanus* that, as a pelagic primary consumer, was analyzed separately from other typically benthic Arthropoda. We used a direct comparison of stable isotope values between paired extracted vs. non-extracted tissues to determine the linear regression correction. Previous work has demonstrated that C:N values can be indicators of lipid content and used to apply mathematical corrections (e.g., Post et al. [70]). We observed small ranges of C:N values despite large ranges of stable isotope values in the test dataset and opted not to incorporate C:N values into the mathematical correction. Moreover, C:N values were not available for all data collated for this analysis.

For calcifying organisms (e.g., Echinodermata), stable isotope values were compared between acidified tissue and lipid-extracted tissue after acidification. Significance level alpha was set at 0.05 and a Bonferroni adjustment was applied for multiple comparisons (Table 2). As a conservative approach, linear regression equations derived from this comparative sample set were used to correct non-lipid extracted samples at the genus level in the rest of the aggregated dataset instead of at higher taxonomic levels. Overall, 13% of data consisting of 53 genera received a mathematical correction using linear regression equations (Tables 2 and S2). On average, after mathematical correction, δ^13^C and δ^15^N values changed by +1.4 ± 1.2‰ and +0.3 ± 0.6‰, respectively, with median changes of +1.1‰ and 0‰.

**Table 2:** Mathematical treatments applied to data to correct stable isotope values for lipid content.

Isotope ratio	Treatment	Taxa	Equation
δ^13^C	HCl + lipid extraction	Arthropoda	y = 0.76x − 3.9
δ^15^N	lipid extraction	Arthropoda	y = 1.02x + 0.04
δ^13^C	HCl + lipid extraction	Bryozoa	y = 0.92x − 1.3
δ^15^N	HCl + lipid extraction	Bryozoa	y = 0.96x + 0.08
δ^13^C	lipid extraction	*Calanus*	y = 1.2x + 7.6
δ^13^C	lipid extraction	Chordata	y = 0.74x − 4.4
δ^15^N	lipid extraction	Chordata	y = 0.66x + 5.0
δ^13^C	HCl + lipid extraction	Cnidaria	y = 1.07x + 2.5
δ^13^C	HCl + lipid extraction	Echinodermata	y = 0.82x − 2.3
δ^15^N	lipid extraction	Echinodermata	y = 1.21x − 0.91
δ^13^C	lipid extraction	Mollusca	y = 0.89x − 1.7
δ^13^C	lipid extraction	Porifera	y = 0.86x − 2.0
δ^15^N	lipid extraction	Porifera	y = 1.36x − 1.79

Only significant relationships are shown. For the ‘lipid extraction’ treatment, paired samples were compared between lipid extraction and no treatment. For the ‘HCl + lipid extraction” treatment, paired samples were compared between acidified samples that were subsequently lipid extracted versus samples only acidified. Alpha was 0.05 with Bonferroni adjustments made for multiple tests. Note that *Calanus* was tested outside of Arthropoda. Genera mathematically corrected within each Phylum are listed in S2 Table.

### Statistical applications

To test the first hypothesis, linear regression analysis was applied to detect temporal changes in stable isotope values. In most cases, data were not normally distributed. While this does not impact linear regression, non-parametric tests were used in subsequent post-hoc tests. We used the non-parametric correlation coefficient Kendall’s tau (τ). Instead of using a one-way ANOVA, a non-parametric Kruskal-Wallis test with a Dunn Test post-hoc pairwise comparison was used. Alpha (α) was set to 0.001 to be conservative and reduce the likelihood of false positives, unless otherwise noted. All analyses and data visualization were performed using R v4.2.2 [72] and RStudio [73]. The map was created using the ‘grfxtools’ package in R, which uses Natural Earth basemap data from the public domain [74].

### Isotopic niche regions (N_R_) and niche overlap

To test the second and third hypotheses, we measured isotopic niches using the approach and tools described by Lysy et al. [75] and Swanson et al. [30] with the R package *nicheROVER* to quantify isotopic niche regions (N_R_) of different groups. As mathematically described by Swanson et al. [30], the method uses a Bayesian framework to estimate the n-dimensional space (N_R_) that a data point has an α probability of being found, where n is the number of isotopes used and α is the user-defined probability, in this case 95%. Then probabilistic niche overlap between two groups is conducted by estimating the probability that a data point randomly selected from one group would fall within the N_R_ space of the other group using a Monte Carlo technique. N_R_ was informed from bivariate δ^13^C and δ^15^N values. The method is robust to differences in sample size, performs well with large sample sizes, and, unlike geometric methods, accounts for species-specific distributions in bivariate space when estimating the probability of overlap [30]. Uncertainty was accounted for by using a Bayesian approach with a noninformative normal-inverse-Wishart prior distribution and 10,000 random permutations to calculate the posterior mean and 95% credible intervals of probabilities of niche overlap. We apply the isotopic niche as a proxy for a trophic niche but recognize they are not synonymous. Both terms are used here with isotopic niche pertaining to stable isotope values, calculated N_R_, and resulting statistical comparison, whereas trophic niche refers to resource use within the ecosystem.

### Investigating potential effects of depth on δ^15^N

A positive relationship between δ^15^N and depth has been documented for benthic deposit and suspension feeders, attributed to biogeochemical alterations to POM particles as they sink through the water column [76,77]. Accordingly, relationships between suspension feeder δ^15^N and depth were investigated using linear regressions for each coastscape independently to ensure results were not affected by variability in depth range among coastscapes. Depth was log_10_-transformed when it improved normality of residuals (shelf and strait coastscapes), and Spearman’s rank correlation analysis was used when assumptions of linear regression were not met (fjord coastscape).

## Results

### Isotopic and temporal patterns of end-members

**Fig 2:**
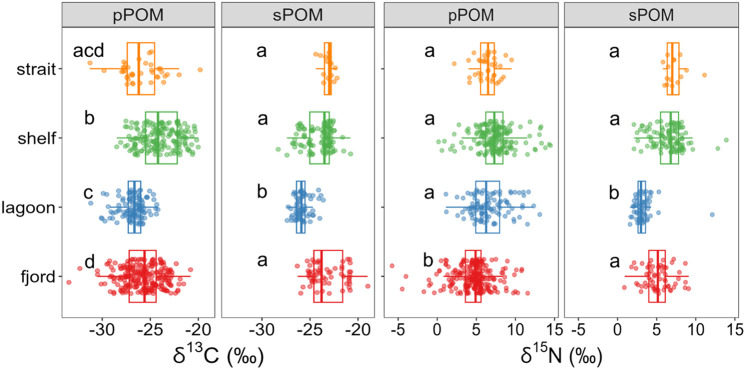
Distribution of stable carbon and nitrogen values for pPOM and sPOM. Data points were plotted over boxplots that summarize distributions. Letters denote significant differences within each column. A Dunn test was used for post-hoc multiple comparisons, and alpha was modified with Bonferonni correction.

δ^13^C and δ^15^N values for pPOM and sPOM exhibited overlap among coastscapes, although there were significant differences among groups (Fig 2). For example, lagoon pPOM and sPOM mostly exhibited ^13^C-depleted values (less than −25‰) compared to other coastscapes. The presence of low δ^15^N values (−5.8 to 11.6‰) of fjord pPOM contributed to their significant difference to all other coastscapes (Table 3). Fjords also exhibited the widest range of δ^13^C values for pPOM (−33.5 to −20.8‰) and the most ^13^C-enriched sPOM value at −19.0‰ (Table 3). Straits exhibited a wide range of δ^13^C values for pPOM that overlapped with other coastscapes, while SPOM from this coastscape was constrained to a narrow range of relatively ^13^C-enriched values (−24.4 to −22.2‰).

**Table 3:** Summary statistics of δ^13^C and δ^15^N for end-members and consumer feeding habits.

			δ^13^C (‰)				δ^15^N (‰)			
Group	coastscape	n	Minimum	Maximum	Median	Mean±s.d.	Minimum	Maximum	Median	Mean±s.d.
pPOM	fjord	237	−33.5	−20.8	−25.6	−25.7 ± 2.0	−5.8	11.6	4.9	4.6 ± 2.4
	lagoon	155	−31.5	−21.7	−26.8	−26.7 ± 1.5	1.1	13.1	6.5	6.7 ± 2.2
	shelf	197	−28.5	−20.0	−24.3	−24.1 ± 2.0	0.2	14.5	7.3	7.1 ± 2.4
	strait	39	−31.3	−19.8	−26.2	−25.9 ± 2.1	2.2	9.5	6.5	6.4 ± 1.4
sPOM	fjord	62	−26.3	−19.0	−23.8	−23.2 ± 1.7	0.9	9.1	5.2	5.1 ± 1.7
	lagoon	101	−28.3	−23.1	−26.0	−25.8 ± 0.9	0.1	12.1	3.1	3.2 ± 1.2
	shelf	126	−28.3	−20.8	−23.6	−24.2 ± 1.5	1.5	13.9	6.6	6.0 ± 2.2
	strait	15	−24.4	−22.2	−23.0	−23.1 ± 0.5	5.8	11.1	7.0	7.2 ± 1.3
iPOM	fjord	11	−30.2	−14.4	−16.7	−19.3 ± 6.2	0.2	5.1	4.8	4.0 ± 1.5
	lagoon	11	−28.4	−25.0	−26.2	−26.3 ± 1.0	5.1	8.6	7.0	6.7 ± 1.0
	shelf	44	−37.0	−5.5	−21.6	−18.6 ± 8.6	3.0	12.5	7.9	7.9 ± 2.1
macroalgae	fjord	57	−37.5	−14.7	−22.7	−25.2 ± 7.0	0.7	13.8	4.3	4.4 ± 2.2
	lagoon	7	−25.1	−21.8	−24.3	−23.9 ± 1.1	2.7	7.2	5.2	5.1 ± 1.4
	shelf	4	−36.9	−21.0	−22.5	−25.7 ± 7.4	5.6	11.4	7.3	7.9 ± 2.7
	strait	12	−27.8	−18.4	−24.6	−24.0 ± 3.0	5.0	14.2	11.5	10.3 ± 3.4
suspension feeder	fjord	629	−30.2	−14.5	−21.9	−22.1 ± 2.1	3.9	18.6	7.8	8.0 ± 2.0
	lagoon	252	−27.9	−13.3	−21.9	−21.9 ± 2.2	4.1	14.7	9.8	9.8 ± 1.9
	shelf	952	−28.1	−13.0	−20.6	−20.6 ± 2.0	3.2	18.2	10.6	10.5 ± 2.1
	strait	154	−26.1	−14.5	−20.5	−20.4 ± 2.6	5.8	19.9	11.3	11.9 ± 3.1
deposit feeder	fjord	400	−26.4	−15.4	−20.9	−20.8 ± 1.7	2.1	16.3	8.7	8.9 ± 2.4
	lagoon	164	−29.3	−15.2	−20.4	−20.2 ± 2.2	6.5	14.0	9.5	9.7 ± 1.5
	shelf	638	−26.7	−13.1	−19.3	−19.6 ± 1.9	3.4	19.4	10.6	10.9 ± 2.5
	strait	58	−24.0	−14.4	−18.9	−19.0 ± 2.1	6.3	15.5	12.2	11.7 ± 2.4
opportunist/scavenger	fjord	289	−28.4	−14.6	−20.1	−20.5 ± 2.4	5.1	17.1	10.3	10.3 ± 1.9
	lagoon	97	−24.4	−14.6	−20.4	−20.4 ± 2.0	1.1	16.6	10.4	10.4 ± 2.8
	shelf	853	−25.8	−11.5	−18.8	−19.2 ± 1.7	0.3	19.2	14.1	13.5 ± 2.1
	strait	94	−26.3	−13.5	−19.0	−19.0 ± 2.6	5.4	16.3	10.6	10.6 ± 2.7
predator	fjord	864	−27.8	−12.9	−20.7	−20.9 ± 2.5	5.5	18.4	11.0	11.1 ± 2.4
	lagoon	318	−28.2	−14.4	−20.6	−20.6 ± 2.5	3.7	21.3	12.4	12.3 ± 2.2
	shelf	1822	−28.0	−12.4	−19.9	−20.0 ± 2.6	5.0	21.2	14.2	13.8 ± 2.0
	strait	141	−27.5	−12.4	−19.5	−19.6 ± 2.6	6.3	19.4	13.8	13.2 ± 3.1

s.d. = standard deviation.

**Fig 3:**
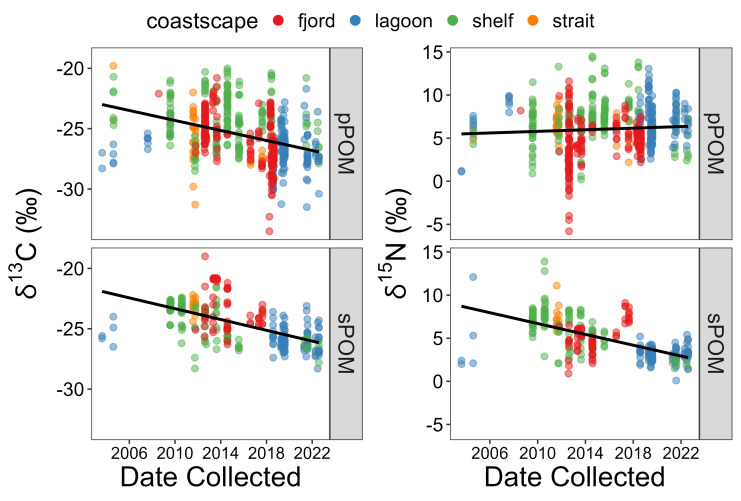
Linear regressions of pPOM and sPOM stable carbon and nitrogen isotope values versus collection date. Color represents the coastscape from which each data point was sampled. See Table 4 for regression statistics. The significant trend between collection date and pPOM δ^13^C (p < 0.001, Kendall’s τ = −0.267) and collection date and sPOM δ^13^C (p < 0.001, Kendall’s τ = −0.446) translate to changes of −2.1 and −2.2 ‰ per decade, respectively, for coastal Arctic end-members. The significant trend between collection date and sPOM δ^15^N (p < 0.001, Kendall’s τ = −0.478) translates to a change of −3.1 ‰ per decade.

The linear regressions between collection date and pPOM δ^13^C values (p < 0.001, Kendall’s τ = −0.267) and collection date and sPOM δ^13^C values (p < 0.001, Kendall’s τ = −0.446) revealed significant negative trends with slopes indicating decadal decreases of −2.1 and −2.2 ‰, respectively (Fig 3; Table 4). Likewise, the trend between collection date and sPOM δ^15^N values (p < 0.001, Kendall’s τ = −0.478) was significantly negative with a decadal change of −3.1 ‰ per decade (Table 4). The linear regression between collection date and pPOM δ^15^N was not significant. To evaluate if these negative trends were influenced by non-uniform coastscape occurrences, specifically lagoons since they bookend the dataset, we excluded the lagoon samples and re-ran the regression analysis. The significant temporal trends described above remained intact and within the same magnitude when lagoon data were excluded (δ^13^C for pPOM: y = −0.000619x − 22.8; δ^13^C for sPOM: y = −0.000618–23.0) versus included (δ^13^C for pPOM: y = −0.000567x – 23.0; δ^13^C for sPOM: y = −0.000613x – 21.9; see Table 4).

**Table 4:** Results from linear regression analysis for δ^13^C or δ^15^N and collection date.

Group	Isotope ratio	Intercept	Slope	Slope p-value	Kendall’s τ	Decadal Δ (‰)
**Endmembers**	**δ** ^**13**^ **C**	−21.9	**−0.000623**	**<0.001**	−0.263	**−2.3**
**Endmembers**	**δ** ^**15**^ **N**	6.8	**−0.000212**	**<0.001**	−0.116	**−0.8**
**pPOM**	**δ** ^**13**^ **C**	−23.0	**−0.000567**	**<0.001**	−0.267	**−2.1**
pPOM	δ^15^N	5.5	0.000127	0.077	0.009	
**sPOM**	**δ** ^**13**^ **C**	−21.9	**−0.000613**	**<0.001**	−0.446	**−2.2**
**sPOM**	**δ** ^**15**^ **N**	8.7	**−0.000861**	**<0.001**	−0.478	**−3.1**
**All consumers**	**δ** ^**13**^ **C**	−19.5	**−0.000168**	**<0.001**	−0.063	**−0.6**
All consumers	δ^15^N	11.6	0.000002	0.910	−0.023	
**Suspension feeders**	**δ** ^**13**^ **C**	−19.6	**−0.000295**	**<0.001**	−0.183	**−1.1**
Suspension feeders	δ^15^N	9.2	0.000099	0.004	0.0197	
**Deposit feeders**	**δ** ^**13**^ **C**	−18.4	**−0.000307**	**<0.001**	−0.168	**−1.1**
Deposit feeders	δ^15^N	10.7	−0.000089	0.059	−0.070	
**Opportunist/scavenger**	**δ** ^**13**^ **C**	−18.2	**−0.000235**	**<0.001**	−0.065	**−0.9**
**Opportunist/scavenger**	**δ** ^**15**^ **N**	10.9	**0.000267**	**<0.001**	0.0759	**+1.0**
Predator	δ^13^C	−20.2	0.000019	0.514	0.0414	
Predator	δ^15^N	12.6	0.000061	0.032	0.0170	

For regression analysis to detect change over time, collection dates were represented as Julian dates with the origin as the earliest sample collected. A Bonferroni adjustment (α/n) was used to conservatively detect significance across 16 tests (i.e., 0.001/16); original p-values are displayed on the table. The non-parametric correlation coefficient Kendall’s τ was used, which is akin to Pearson’s r. Significant slopes according to the Bonferroni adjustment are bolded. Decadal change (Δ) was calculated by multiplying the slope, which was determined using the time unit day, by the number of days in 10 years, or 3650. Only significant Decadal Δ are shown.

### Isotopic and temporal patterns of consumers

**Fig 4:**
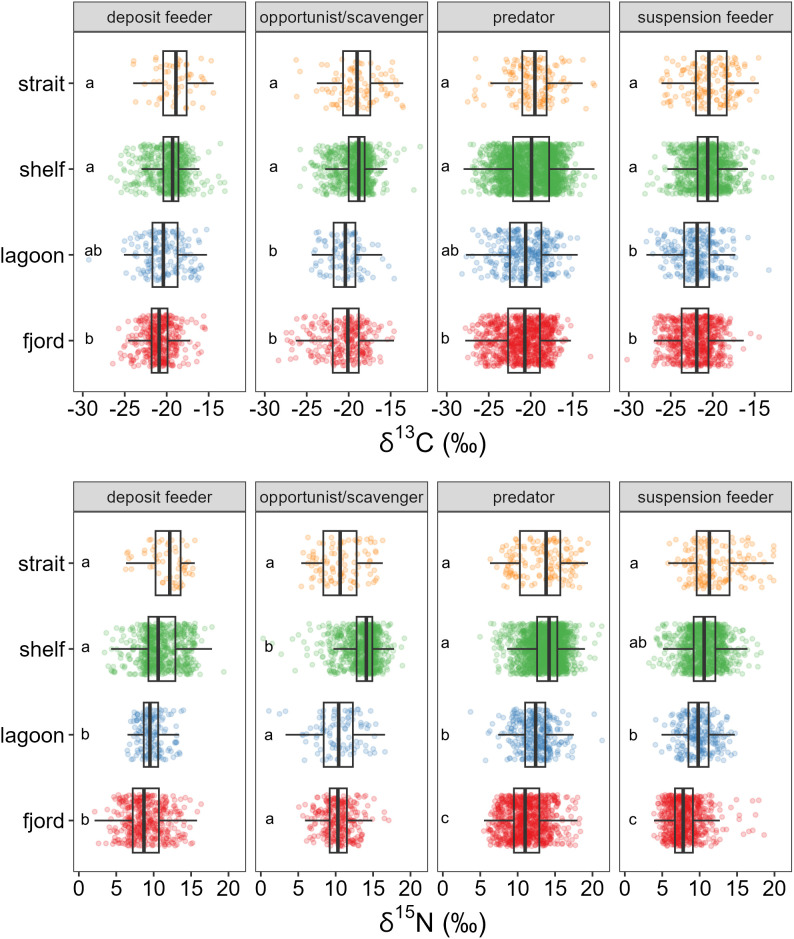
Distribution of stable isotope values for consumers faceted by coastscape and feeding habit. Significant differences denoted with letters within each facet. A Dunn test was used for post-hoc multiple comparisons, and alpha was modified with Bonferonni correction. Note different x-axes.

Overall δ^13^C values ranged from −30.2 to −11.5‰, and δ^15^N values ranged from 0.3 to 21.3‰. The isotopic centroid for the entire dataset was −20.4 and 11.6‰ for δ^13^C and δ^15^N, respectively. While stable isotope value ranges overlapped among all feeding groups, statistical differences existed between some groups. For δ^13^C in all feeding modes, there were no significant differences between straits and shelves, or between lagoons and fjords (Fig 4). Median δ^13^C values for consumers from lagoons and fjords were significantly different and more ^13^C-depleted than those in shelves or straits.

Patterns in δ^15^N values for consumers were similar to those in δ^13^C, except for the opportunist/scavenger group. Overall, δ^15^N values were overlapping among coastscapes for each feeding habit, although significant differences were detectable among groups (Fig 4). Median values of lagoon and fjord consumers were generally more ^15^N-depleted than straits and shelves except for opportunist/scavengers from straits, which were also ^15^N-depleted compared to the shelf counterparts (Fig 4).

Over the period represented by this dataset (~ 2 decades), the linear regression between collection date and all consumer δ^13^C values was significant and negative (Table 4; S1 Fig). Specifically, the suspension feeder, deposit feeder, and opportunist/scavenger groups exhibited negative linear regressions between collection date and δ^13^C values, with slopes that extrapolated to respective changes of −1.1‰, −1.1‰, and −0.9‰ per decade (Table 4). The linear regression between δ^15^N and time was not significant when using all consumers in the dataset. Only the opportunist/scavenger feeding habit showed a significant linear regression between collection date and δ^15^N values.

Mean posterior estimates of niche overlap were generally high across coastscapes (Table 5; S2–S5 Figs), mostly resulting in an estimated >70% niche region space (N_R_) overlap. As an exception to the trend, opportunist/scavengers from shelves exhibited ~50% overlap with other coastscapes. This coincides with significantly higher δ^15^N values for opportunist/scavengers in shelves than those in fjords, straits, or lagoons (Fig 4).

**Table 5:** Quantification of isotopic niche overlap for feeding habits between coastscapes.

	fjord	lagoon	shelf	strait
**Predator**				
fjord	NA	93.3 (90.2, 95.9)	72.4 (67.0, 75.9)	94.5 (90.9, 97.4)
lagoon	86.4 (82.1, 90.1)	NA	81.2 (76.6, 85.4)	91.5 (86.6, 95.7)
shelf	80.4 (76.7, 83.8)	92.2 (88.5, 95.3)	NA	94.0 (90.4, 96.9)
strait	80.6 (74.2, 85.9)	86.3 (80.0, 91.7)	80.5 (74.6, 85.7)	NA
**Opportunist/scavenger**				
fjord	NA	91.3 (83.8, 97.1)	55.6 (49.6, 61.5)	95.6 (91.1, 98.5)
lagoon	76.2 (65.1, 85.7)	NA	48.7 (37.4, 59.6)	84.27 (73.0, 93.4)
shelf	70.1 (62.1, 78.0)	94.3 (84.3, 99.2)	NA	94.2 (87.6, 98.5)
strait	80.5 (72.4, 87.6)	82.3 (71.3, 92.3)	51.9 (43.9, 60.2)	NA
**Deposit feeder**				
fjord	NA	77.5 (69.2, 85.5)	87.4 (83.4, 91.1)	83.3 (71.4, 93.0)
lagoon	91.9 (85.8, 96.2)	NA	91.9 (85.9, 96.2)	92.5 (84.0, 98.0)
shelf	87.9 (83.8, 91.5)	75.0 (66.6, 83.4)	NA	94.4 (89.4, 98.0)
strait	73.7 (62.8, 83.1)	62.6 (49.9, 75.6)	88.3 (80.5, 94.1)	NA
**Suspension feeder**				
fjord	NA	93.1 (88.6, 96.6)	73.4 (68.7, 78.0)	89.2 (82.8, 95.0)
lagoon	89.4 (84.2, 93.6)	NA	79.3 (72.7, 85.3)	92.3 (86.2, 96.7)
shelf	79.1 (74.4, 83.7)	91.8 (86.3, 96.2)	NA	98.4 (96.9, 99.5)
strait	53.9 (46.7, 60.8)	68.0 (58.3, 77.4)	74.5 (68.1, 80.1)	NA

Values are mean posterior estimates of niche region (N_R_) overlap probability. Values in parentheses are the 95% credible interval (CI) range (2.5%, 97.5%) for consumers in each feeding habit between coastscapes. Matrix should be read as the probability that an individual from the coastscape indicated in the row will be found within the isotopic niche indicated in the column.

### Comparison of fjord and shelf isotopic niches across sectors

**Fig 5:**
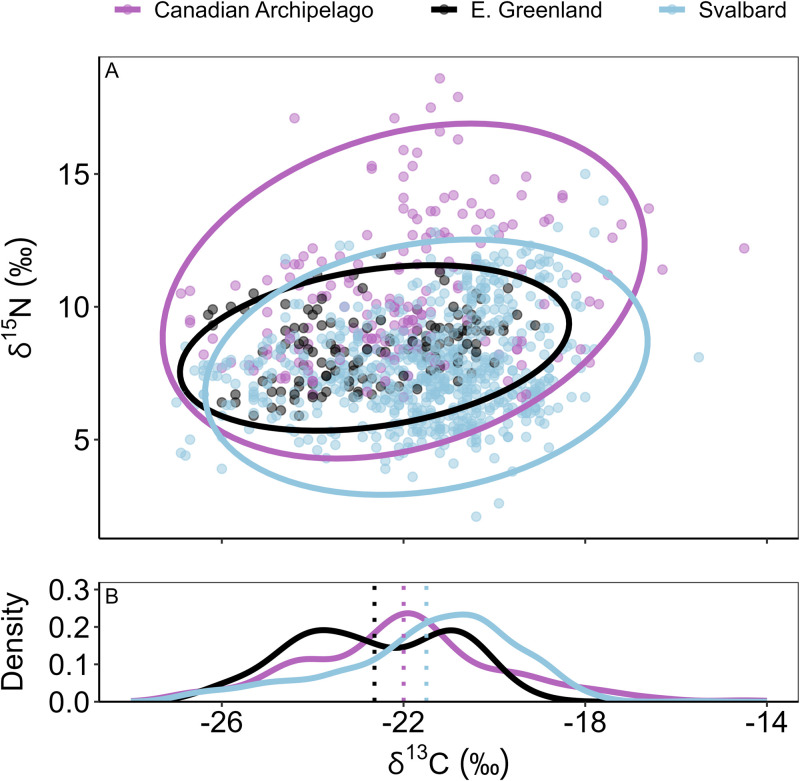
Niche overlap within fjord communities. (A) Mean posterior estimates of isotopic niche regions constructed from δ^15^N and δ^13^C values for benthic deposit and suspension feeders sampled in fjord coastscapes, in three geographic sectors. Niche regions were calculated at alpha = 0.95. (B) Density distribution of δ^13^C data from each sector, with dashed lines indicating the sample mean.

The deposit and suspension feeders sampled in fjord coastscapes exhibited greater among-sector variability along the δ^15^N axis than along the δ^13^C axis (Fig 5). Notably, there was a high probability of niche overlap (> 96%) estimated for East Greenland consumers in both the Canadian Arctic Archipelago and Svalbard isotopic niches (Table 6). Consumers sampled in the Canadian Arctic Archipelago had the largest δ^15^N and δ^13^C ranges (Fig 5; Table 6); thus, the probabilities of niche overlap of consumers from Svalbard or East Greenland falling into the isotopic niche of Canadian Archipelago consumers was relatively high (Table 6). Conversely, Canadian Archipelago consumers had a lower probability of falling into the isotopic niches of the other two sectors that exhibited smaller N_R_.

**Table 6:** Quantification of isotopic niche overlap in fjords.

FJORD	Canadian Archipelago	E. Greenland	Svalbard
**Canadian Archipelago**	NA	52.6 (44.8, 60.9)	66.4 (59.8, 73.0)
**E. Greenland**	98.7 (96.7, 99.7)	NA	96.8 (94.0, 98.6)
**Svalbard**	87.4 (80.7, 93.0)	70.5 (63.2, 77.6)	NA

Mean posterior estimates of overlap probability and 95% credible interval (CI) range for suspension and deposit feeders from each sector within fjords. Numbers represent the probability that an individual from the sector indicated in the row will be found within the niche indicated in the column. Values are reported as mean posterior estimate with lower and upper limits in parentheses (2.5% CI, 97.5% CI).

**Fig 6:**
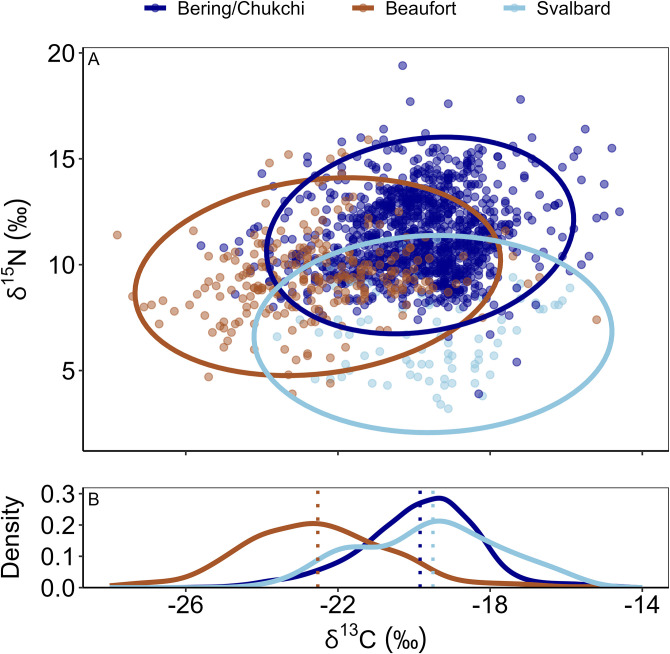
Niche overlap within shelf communities. (A) Mean posterior estimates of isotopic niche regions constructed from δ^15^N and δ^13^C values for benthic deposit and suspension feeders sampled in shelf coastscapes, in three geographic sectors. Niche regions were calculated at alpha = 0.95. (B) Density distribution of δ^13^C data from each sector, with dashed lines indicating the sample mean.

In contrast, the deposit and suspension feeders sampled in shelf coastscapes exhibited among-sector variability along the δ^13^C and δ^15^N axes (Fig 6). However, the Beaufort Sea and Northern Bering/Chukchi Seas shelf consumers exhibited a higher probability of overlap than either did with shelf consumers from the Svalbard shelf (Table 7), attributable mostly to lower mean δ^15^N of the Svalbard shelf niche.

**Table 7:** Quantification of isotopic niche overlap in shelves.

SHELF	Bering/Chukchi	Beaufort	Svalbard
**Bering/Chukchi**	NA	77.6 (69.8, 84.8)	42.6 (28.2, 60.3)
**Beaufort**	67.8 (61.8, 73.7)	NA	52.5 (36.9, 70.0)
**Svalbard**	37.7 (28.8, 47.2)	49.0 (36.6, 61.5)	NA

Mean posterior estimates of overlap probability and 95% credible interval (CI) range for suspension and deposit feeders from each sector within shelves. Numbers represent the probability that an individual from the sector indicated in the row will be found within the niche indicated in the column. Values are reported as mean posterior estimate with lower and upper limits in parentheses (2.5% CI, 97.5% CI).

### Investigating potential effects of depth on δ^15^N

Linear relationships between benthic deposit and suspension feeder δ^15^N and depth were not significant for lagoons, fjords, or shelves. There was a significant, but weak positive correlation between deposit and suspension feeder δ^15^N and log_10_ depth for straits (depth range 17–789 m; Spearman’s correlation, *rho* = 0.17, *p* < 0.001).

## Discussion

### Temporal changes in stable isotope values

Contrary to the null hypothesis that stable isotope values of end-members would not change over a decadal scale, pPOM and sPOM became 2.1‰ and 2.2‰ more ^13^C-depleted per decade, respectively, from 2002–2022 across Arctic coastal environments. This aligns with results from de la Vega et al. [35] who found a ^13^C-depletion of ~1.5‰ per decade for pPOM across the Arctic basin and adjacent shelf seas. Those authors attributed the trend, in part, to the Suess effect, which ascribes the decreasing δ^13^C value of atmospheric CO_2_ to the emissions from ^13^C-depleted fossil fuels [78]. The atmospheric CO_2_ signature becomes reflected in the δ^13^C value of dissolved inorganic carbon (DIC) in marine waters and, subsequently, by primary producers after carbon fixation [79]. With their pan-Arctic assessment, de la Vega et al. [35] showed DIC became ~0.1‰ more ^13^C-depleted per decade between 1977–2014 due to the Suess effect. However, that rate of change for DIC was an order of magnitude less than the ~ 2‰ decadal change in pPOM and sPOM found by this study; therefore, the Suess effect can only partially explain the change in the Arctic primary producers observed in this study.

As suggested by de la Vega et al. [35], one explanation for these end-members becoming more ^13^C-depleted over time is a change in the relative composition of pPOM and sPOM, since they are only operationally defined and consist of a mixture of organic matter of varied origin. A decreased contribution of sea ice algae [35], which are typically ^13^C-enriched compared to phytoplankton [80], increased contributions of ^13^C-depleted terrestrially-derived organic matter [15], or both, would account for the observed isotopic changes in pPOM and sPOM. Greater terrestrial inputs to coastal pPOM and sPOM are plausible given the increase in river discharge, thawing permafrost, and coastal erosion [5,7,8]. Freshwater aquatic microalgae, with δ^13^C values of −33.1 ± 4.7‰, constitute 39–60% of the POM exported by rivers to coastal environments [18]. Increased freshwater export of this ^13^C-depleted end-member could also contribute to the decreasing pPOM and sPOM values in coastal Arctic ecosystems. It is also possible that increased inputs of riverine DIC and/or DIC remineralized from terrestrial organic matter in coastal habitats would cause ^13^C-depletion of the DIC pool [81]. With the baseline stable carbon isotope value for primary producers left-shifted, the autochthonous microalgal components of the pPOM and sPOM pools would become ^13^C-depleted over time as well.

The similar temporal trends of δ^13^C exhibited by the pPOM and sPOM pools appear to support the tight coupling between the two pools often observed in the Arctic [64,65,82–86]. Therefore, it was unexpected that the δ^15^N values for the two pools would exhibit different temporal trends (Fig 3). One explanation for no temporal trend for δ^15^N in pPOM might stem from the timeframe over which it forms and the inorganic nitrogen sources it assimilates compared to the sPOM pool. As the living microalgal component of pPOM blooms during the short Arctic growing season, it would assimilate and reflect the δ^15^N signature of its inorganic nitrogen pool, typically marine nitrate. Coastal Arctic marine nitrate δ^15^N values typically range between 5–8‰ [87], and the mean and median values from pPOM in this dataset fall within that range at 6.1 and 6.0‰, respectively. Therefore, we may be observing predominant usage of marine nitrate by coastal primary producers in the pPOM pool over the past two decades, despite recent findings that terrestrial nitrogen fuels about one-third of Arctic primary production [17]. Conversely, sPOM exhibited the trend of ^15^N-depletion over time, suggesting over the two decades analyzed here its association with marine nitrate weakened while its association with terrestrial nitrogen sources strengthened. Alternatively, the sPOM δ^15^N trend might not be related to microalgal assimilation of nitrate, but rather a larger proportion of ^15^N-depleted allochthonous terrestrial OM entering the sPOM pool that is not retained in pPOM. This seems plausible since coastal sediments are repositories of organic matter [88] and accumulate over long periods of time compared to the water column, which constantly refreshes with new microalgal growth. Interestingly, terrestrial OM can remain suspended compared to marine particles that sink faster and contribute more to sPOM over millennial timescales [89], so terrestrial OM deposition near the coast cannot be broadly assumed, especially in deeper zones. Still, biomarker evidence demonstrates that high proportions of sPOM in the coastal Arctic are derived from terrestrial sources [90,91]. Furthermore, it has been noted in lagoons [15], shelves [32,48,58], and fjords [45] that sPOM was consistently 0.4 to 4.0‰ more ^15^N-depleted than pPOM.

Consumers of pPOM and sPOM also reflected a ^13^C-depletion trend over time. While a significant trend was found with all pooled consumers, this was driven by suspension feeders, deposit feeders, and opportunist/scavengers (Table 4). The regression slope for those three groups extrapolated to ~ −1‰ per decade for δ^13^C, which interestingly was less change than what was detected in the end-members. A similar pattern was also detected by de la Vega et al. [35], who measured significant ^13^C-depletion for marine mammals over decadal timescales but at a lesser rate than pPOM was changing. This may indicate that benthic invertebrates preferentially select for organic matter sources with more nutritional quality [92]. Thus, increasing inputs of terrestrial organic matter in end-member pools are not reflected to the same extent in the diet of benthic invertebrates because of the low nutritional quality of terrestrial organic matter.

As a caveat, we acknowledge no single end-member or consumer group is necessarily changing by the rates indicated by the regression slopes of pooled samples. These trends detected the overall change of aggregated data across the entire Arctic coastal areas as our intent was to examine coarse, pan-Arctic patterns.

### Isotopic niche overlap

The second hypothesis that organic matter sources would be differentially assimilated by Arctic consumers based on geomorphological differences between coastscapes was not supported. Consumers of the same feeding habit between coastscapes exhibited high isotopic niche overlap (Table 5). Previous examples have shown that different feeding habits can share trophic niches within specific Arctic coastscapes, for example, within Arctic lagoon systems [15,93], on the Pacific Arctic shelf [32,94], in strait regions of the Canadian Arctic Archipelago [95,96], and in Arctic fjords [60,66]. Most commonly, this high trophic niche overlap is attributed to the high trophic plasticity of many Arctic invertebrate consumers that allows them to capitalize on a wide variety of food sources, a strategy that is considered adaptive due to the high seasonality of primary production in an Arctic system [46]. However, we found that this overlap in isotopic niche space applies not only to feeding habits within a coastscape but also across geomorphologically very different coastscapes.

Evidence to refute the third hypothesis that isotopic niches for a common coastscape across longitudinal sectors would overlap was less clear. Although we generally found overlap in the isotopic niches between common coastscapes, we also found some regional distinctions. Fjords in the Canadian Arctic Archipelago, Eastern Greenland, and Svalbard exhibited overlapping isotopic niches on the δ^13^C axis, indicating mostly isotopically similar food sources were assimilated by consumers (Fig 5). However, the isotopic niche for consumers from the Canadian Arctic Archipelago extended to relatively ^15^N-enriched space, unlike those from Eastern Greenland and Svalbard. The greater depth in some of the Canadian Arctic Archipelago stations can facilitate depth-associated ^15^N-enrichment of sinking pPOM [61], expanding the isotopic niche compared to its counterparts.

While isotopic niche overlap occurred between suspension and deposit feeders in the Northern Bering/Chukchi Seas, Beaufort Sea, and Svalbard sectors, each isotopic niche extended into distinct isospace for both carbon and nitrogen (Fig 6). The distinctly ^13^C-depleted values in the Beaufort Sea (Fig 6; Table 7) align with the enormous export of terrestrial carbon originating from the Mackenzie River in the eastern Beaufort and the Colville River in the western Beaufort [97] and inputs from the Alaska Coastal Water from the Chukchi Sea [32,98]. Evidence for consumer assimilation of these ^13^C-depleted terrestrial sources by consumers has been widely noted [34,48,99]; thus, our finding that the Beaufort Sea consumer isotopic niche extends into ^13^C-depleted isospace compared to other longitudinal sectors is not surprising. Conversely, the suspension and deposit feeders from the Svalbard shelves had δ^15^N values that were more ^15^N-depleted compared to the Northern Bering/Chukchi Seas and Beaufort Sea sectors (Fig 6). These lower δ^15^N values for shelf consumers were also reflected in pPOM (Table 3); mean δ^15^N values for Northern Bering/Chukchi Seas (7.6 ± 2.4‰) and Beaufort Sea (6.8 ± 2.2‰) sector pPOM were ~ 3‰ higher than in the Svalbard (4.4 ± 2.5‰). We surmise this difference in pPOM δ^15^N values is ultimately related to different sources of dissolved inorganic nitrogen assimilated by primary producers that comprise the particulate pools in these two regions. For example, upwelled nitrate from the Pacific Basin that flows northward to the Northern Bering and Chukchi Seas is ^15^N-enriched on average by 3‰ compared to relatively ^15^N-depleted nitrate that is delivered to the waters surrounding Svalbard from the Atlantic Basin [100]. This trend may be exacerbated in the coastal waters around Svalbard by ongoing “Atlantification”, a term describing the increased influence of Atlantic Ocean heat and nutrients in the Atlantic Arctic region due to massive reduction in sea ice and a weakened halocline [101]. The δ^15^N signature of the Beaufort Sea sector west of the Mackenzie River outflow is more intermediate, a reflection of both terrestrial inorganic-N sources from the Alaska Coastal Water and the advection of ^15^N enriched pPOM from Northern Bering Sea water that is advected eastward onto the Alaskan Beaufort Sea shelf as the Beaufort Undercurrent [98]. Northern Bering/Chukchi Seas sector pPOM was the most ^15^N-enriched of any sector, primarily derived from the aforementioned Bering Sea water.

### Organic matter resources assimilated in Arctic coastscapes

Following the tenets of stable isotope ecology, one should be able to infer the range of assimilated organic matter sources by the range of stable isotope values exhibited by consumers [24]. All feeding groups among pan-Arctic coastscapes contained consumers with δ^13^C values between −24.0‰ and −15.4‰ (Table 3). The large δ^13^C ranges for each of the four feeding groups among the four coastscapes (> 9‰) is a clear indicator of assimilation of multiple isotopically-distinct organic matter sources across the coastal Arctic.

Like consumers, pPOM and sPOM from the four coastscapes collectively reflected large δ^13^C ranges: 12.7‰ (−33.5‰ to −20.8‰) in fjords; 9.8‰ (−31.5‰ to −21.7‰) in lagoons; 8.5‰ (−28.5‰ to −20.0‰) on shelves; and 11.5‰ (−31.3‰ to −19.8‰) in straits (Fig 2, Table 3). The wide range of δ^13^C values for pPOM and sPOM indicated these end-member pools were indeed a conglomeration of multiple isotopically-distinct organic matter sources.

Phytoplankton are a common constituent of the pPOM mixture in most marine habitats and may sink and become incorporated into the sPOM pool [64,65,85]. Within the pPOM aggregation in this dataset, data rows specifically called phytoplankton ranged from −26.7‰ to −20.3‰ for δ^13^C. This matches other reported values for coastal Arctic phytoplankton of −26‰ to −19‰ [92,93,102]. However, the range of phytoplankton stable isotope values (−26‰ to −19‰) cannot solely account for the range of pPOM (−33.5‰ to −19.8‰) and sPOM (−28.5‰ to −19.0‰) reported here. Therefore, other ^13^C-depleted carbon sources relative to phytoplankton must have contributed to the mixture of POM pools.

Potential sources typically ^13^C-depleted compared to marine phytoplankton include terrestrial plant material (−27.7 ± 1.3‰) [18], thawed permafrost organic matter (−27.7 ± 1.4‰) [18], freshwater microalgae (−33.1 ± 4.7‰) [18], red macroalgae (−33.0 ± 3.3‰) including common Arctic genera such as *Phycodrys*, *Polysiphonia*, and *Odonthalia* [15,18,60,103], and some brown macroalgae such as *Desmarestia* (<−27‰) [60]. Organic matter sources in Arctic coastscapes that can be ^13^C-enriched compared to phytoplankton are brown macroalgae like the kelp *Laminaria* and other macroalgae including *Fucus, Pylaiella, and Ectocarpus* (−22‰ to −14‰) [51,60,104], iPOM [80,92,96,105–107], and benthic microalgae [108–112].

The coastscapes that exhibit the required hard substrate for significant macroalgal production likely exist east of the Beaufort Sea in the straits and fjords of the Canadian Arctic Archipelago, Greenland, and Svalbard. Red and brown macroalgae require hard benthic substrate (e.g., rocky shorelines, seafloor cobbles, etc.) and sufficient benthic PAR to subsist. Lantuit et al. [2] report that approximately one-third of the Arctic coastline is lithified. In addition, coastal areas impacted by rivers receive high levels of sediment inputs. Macroalgal carbon assimilation is less prevalent than other available sources where freshwater influence is pronounced [55,60].

**Fig 7:**
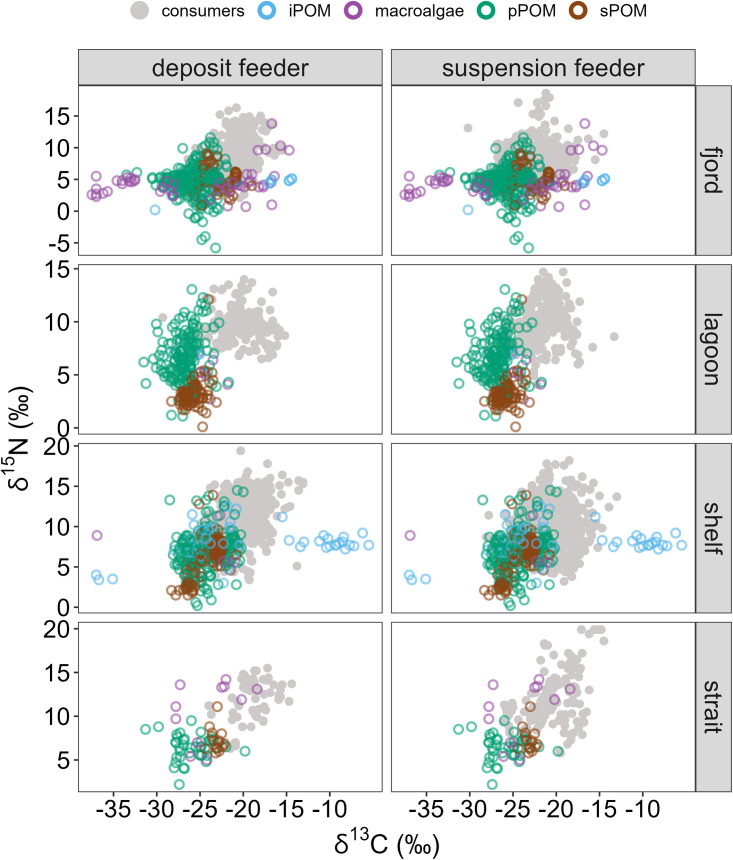
Stable isotope biplots of end-members (colored points) faceted by coastscape and overlayed consumers (gray points). Panels are faceted by feeding habit (deposit feeder or suspension feeder) and coastscape. Deposit and suspension feeders were plotted to depict primary consumers. Note the different y-axis scale for fjords. Predators and scavenger/omnivore data not shown.

iPOM carbon is a critical resource for Arctic consumers [63,113,114]. One recent pan-Arctic biomarker analysis demonstrated that not only was iPOM a carbon subsidy available year-round in sediment food banks, but most (133 of 155) species analyzed, including invertebrates and vertebrates, assimilated iPOM carbon to some degree [115]. Another study using the same biomarkers demonstrated fjord and shelf consumers near Young Sound, Greenland assimilated no less than 60% and oftentimes more than 90% iPOM-derived organic matter [83]. While this trend likely prevails throughout Arctic coastscapes, the stable isotopic evidence for its assimilation is not always clear (Fig 7). In some cases, for example on shelves, the ^13^C-enriched values of iPOM may explain some of the ^13^C-enriched values of consumers. Within the dataset assembled for this study, iPOM possessed δ^13^C values as high as −5.5‰ in some instances, but in other cases iPOM data were less ^13^C-enriched than phytoplankton (Fig 7). iPOM δ^13^C values ranged from −30.2‰ to −14.4‰ in fjords, from −28.4‰ to −25.0‰ in lagoons, and −37.0‰ to −5.5‰ on shelves (Table 3). The wide range in δ^13^C values is attributed to a variety of factors including PAR availability, temperature, δ^13^C-DIC, DIC limitation, nutrient concentration, and cell growth rates [116,117]. However, iPOM δ^13^C values observed in this dataset were not only variable but also overlapped with other organic matter sources in lagoons, fjords, and shelves, so natural abundance stable isotopes were too confounded to demonstrate the same widespread assimilation reported elsewhere [83,115].

Benthic microalgal carbon is recognized as a major source of labile carbon to consumers in non-Arctic coastal habitats, such as fjord-like areas in Antarctica [111], shallow temperate bays [109], and shallow continental shelves [108]. The shallow coastal habitats of the Arctic where PAR can penetrate to the seafloor [22] can foster populations of benthic microalgae, which would be a food source to the benthic food web [15,58,112,118]. These algae are known for their high relative contribution to total primary production rates in Arctic waters under 30 m depth compared to phytoplankton and ice algae [119] and can be observed through high sediment pigment concentrations [85,120–122], but direct measurement of stable isotope values for Arctic benthic microalgae remains elusive [58]. Modeled values of benthic microalgae δ^13^C values for the Beaufort Sea shelf are reported to lie between −24‰ and −18‰ [110], which falls within the range of a potential food source for shelf consumer compiled in this study (Figs 4 and 7).

^13^C-enriched benthic primary producers like macroalgae, benthic microalgae, and ice algae contribute up to 30% of annual phytoplankton production at a pan-Arctic Ocean scale based on a light availability model [23], and may be proportionally more important in shallow coastal areas [120]. Deeper Arctic coastscapes like the straits of the Canadian Arctic Archipelago and deep fjords would not host benthic microalgae unless they were physically advected from shallow depths.

We acknowledge that the use of bulk stable isotopes, broad feeding habit categories, and wide geographic coverage leaves open the possibility that species-specific selective assimilation, isotopic routing between tissue types, and differences in isotopic discrimination could occur without detection by the methods employed here [123–125]. However, we argue the comparisons of bulk stable isotope values between consumers and organic matter sources described above are robust to individual or species-level instances of atypical stable isotope behavior due to the volume of aggregated data used to parse broad patterns.

### Missing pieces to the trophic puzzle

There still remain some gaps in our understanding of isotopic relationships and the relative importance of sampled end-members. While our evidence suggests that POM pools are becoming more ^13^C-depleted over decadal timescales (Fig 3), there are many consumers that exist in ^13^C-enriched isospace without an obvious connection to a ^13^C-enriched end-member (Fig 7). Typically, trophic enrichment factors of ~+1‰ are assumed for δ^13^C and +1–4‰ for δ^15^N [126]. Nadon and Himmelman [127] prescribed trophic enrichment factors of +4‰ for δ^13^C between primary producers and primary consumers based on their observations, but even with this increased trophic step the end-members represented here, particularly in lagoons and straits, cannot account for the ^13^C-enriched values of consumers. Notably, the few iPOM values reported for lagoons were relatively ^13^C-depleted, and no iPOM data were reported for straits. We note the lack of data imported during this non-exhaustive literature survey is not evidence of absence for iPOM in these coastscapes, especially as sophisticated biomarker methods continually demonstrate the importance of iPOM to consumers [83,115]. Some iPOM stable isotope data from the Canadian Arctic Archipelago that failed to satisfy the metadata requirements for inclusion in this study demonstrate iPOM can be a ^13^C-enriched food source to the benthic food web [96,106,107]. The ^13^C-depleted values of iPOM in lagoons is puzzling but not out of the range of other reported values [35,105,106]. ^13^C-enrichment of sea ice algae often occurs within assemblages with high chlorophyll *a* concentrations and may occur later in the season when more light availability increases primary production rates [105,106].

Moreover, there could be a mismatch between end-member sampling and the isotopic turnover window reflected by the consumers if both are collected concurrently. The vast majority of samples represented in this pan-Arctic study were collected between mid-July and mid-September (S6 Fig). Since the isotopic turnover of Arctic invertebrates [128,129] and fish [130] can be quite slow, on the order of 1–2 months if not longer, some consumers collected during summer months may still isotopically reflect their diet from previous ice-covered months, like iPOM.

Lastly, it is unclear what role bacteria play in modifying organic matter sources prior to assimilation by the benthic food web or act as a food source themselves. Sinking organic matter has been documented to undergo stable isotope value changes from microbial breakdown before reaching the benthos [61,77]. This suggests organic matter would continue to undergo microbial breakdown once it arrives to the seafloor. Bacterial breakdown of organic matter has been invoked as a mechanism that results in an unmeasured ^13^C-enriched food source for benthic consumers that exhibit δ^13^C values heavier than sampled end-members in Arctic food webs [57,131,132]. Techniques more sophisticated than bulk stable isotope analysis are required to follow bacterially-derived carbon into the food web, and this approach indicates it is significantly important to Arctic consumers [133].

## Conclusion

This analysis of Arctic coastal stable isotope data from 1999–2022 showed long-term changes in food sources (δ^13^C values of pPOM and sPOM) that may reveal the effects of warming trends across coastal Arctic ecosystems. The δ^13^C values of pPOM and sPOM becoming more ^13^C-depleted over the last two decades can be partially related to the Suess Effect where primary producers incorporate ^13^C-depleted CO_2_ derived from fossil fuel combustion. However, these isotopic temporal trends of the POM pools are most likely based on a combination of (1) increased incorporation of terrestrially-derived organic matter, (2) increased incorporation of autochthonous microalgae using ^13^C-depleted terrestrially-derived DIC, or (3) decreased contributions from ^13^C-enriched sympagic sources. This ^13^C-depletion over two decades was also found in Arctic consumers. Across all Arctic coastscapes, consumers exhibited overlapping isotopic composition, notably with wide δ^13^C ranges that indicated assimilation of multiple organic matter sources, including terrestrial organic matter, pPOM, sPOM, iPOM, macroalgae, and probably benthic microalgae except in the deep Canadian straits. This consistent pattern across coastscapes supports the notion that Arctic benthic invertebrate consumers have high trophic plasticity that allows them to use a wide variety of sources, which stabilizes food webs [56,134,135]. Lastly, consumers within the same coastscape exhibited overlapping isotopic niches between longitudinal sectors, with a few notable exceptions. Consumer isotopic niches reflected regional trends in freshwater and nutrient inputs that influence endmember isotope ranges, for example, the signature of increasing freshwater influence in the Beaufort Sea and the “Atlantification” near Svalbard represent regional effects of climate warming visible in the pan-Arctic isoscape.

## Supporting information

S1 Fig Linear regressions of consumer stable carbon and nitrogen isotope values and collection date. Graphical display of regressions faceted by feeding habit and stable isotope for the linear regressions presented in Table 4.
(PNG)

S2 Fig Posterior estimates of niche overlap for suspension feeders. Probability that an individual sampled in the coastscape listed for the row would also occur within the isotopic niche of the same feeding guild samples in the coastscape listed for the column.
(TIF)

S3 Fig Posterior estimates of niche overlap for deposit feeders. Probability that an individual sampled in the coastscape listed for the row would also occur within the isotopic niche of the same feeding guild samples in the coastscape listed for the column.
(TIF)

S4 Fig Posterior estimates of niche overlap for opportunist/scavengers. Probability that an individual sampled in the coastscape listed for the row would also occur within the isotopic niche of the same feeding guild samples in the coastscape listed for the column.
(TIF)

S5 Fig Posterior estimates of niche overlap for predators. Probability that an individual sampled in the coastscape listed for the row would also occur within the isotopic niche of the same feeding guild samples in the coastscape listed for the column.
(TIF)

S6 Fig Histogram of day-of-year for sample collection for data collected for this meta-analysis. Counts represent rows of data. End-members and consumers from all years included.
(PNG)

S1 Table Data sources used for meta-analysis. Full bibliographic information available for each publication listed in References. Projects listed with acronyms represent datasets unpublished in peer-review literature. All data are available in public repository referenced in manuscript.
(DOCX)

S2 Table Genera in the meta-analysis dataset that were mathematically corrected for lipid content. Corrections were from equations represented in Table 2.
(DOCX)

## References

[1] Jakobsson M. Hypsometry and volume of the Arctic Ocean and its constituent seas: Hypsometry and volume of Arctic Ocean. Geochem Geophys Geosyst. 2002;3(5):1–18. doi: 10.1029/2001gc000302

[2] Lantuit H, Overduin PP, Couture N, Wetterich S, Aré F, Atkinson D, et al. The Arctic Coastal Dynamics Database: A New Classification Scheme and Statistics on Arctic Permafrost Coastlines. Estuaries and Coasts. 2011;35(2):383–400. doi: 10.1007/s12237-010-9362-6

[3] Rantanen M, Karpechko AYu, Lipponen A, Nordling K, Hyvärinen O, Ruosteenoja K, et al. The Arctic has warmed nearly four times faster than the globe since 1979. Commun Earth Environ. 2022;3(1). doi: 10.1038/s43247-022-00498-3

[4] Haine TWN, Curry B, Gerdes R, Hansen E, Karcher M, Lee C, et al. Arctic freshwater export: Status, mechanisms, and prospects. Global and Planetary Change. 2015;125:13–35. doi: 10.1016/j.gloplacha.2014.11.013

[5] Stadnyk TA, Tefs A, Broesky M, Déry SJ, Myers PG, Ridenour NA, et al. Changing freshwater contributions to the Arctic: A 90-year trend analysis (1981–2070). Elementa: Science of the Anthropocene. 2021;9(1). doi: 10.1525/elementa.2020.00098

[6] Rawlins MA, Karmalkar AV. Regime shifts in Arctic terrestrial hydrology manifested from impacts of climate warming. The Cryosphere. 2024;18(3):1033–52. doi: 10.5194/tc-18-1033-2024

[7] Schuur EAG, Abbott BW, Commane R, Ernakovich J, Euskirchen E, Hugelius G, et al. Permafrost and Climate Change: Carbon Cycle Feedbacks From the Warming Arctic. Annu Rev Environ Resour. 2022;47(1):343–71. doi: 10.1146/annurev-environ-012220-011847

[8] Nielsen DM, Pieper P, Barkhordarian A, Overduin P, Ilyina T, Brovkin V, et al. Increase in Arctic coastal erosion and its sensitivity to warming in the twenty-first century. Nat Clim Chang. 2022;12(3):263–70. doi: 10.1038/s41558-022-01281-0

[9] Cai Q, Wang J, Beletsky D, Overland J, Ikeda M, Wan L. Accelerated decline of summer Arctic sea ice during 1850–2017 and the amplified Arctic warming during the recent decades. Environ Res Lett. 2021;16(3):034015. doi: 10.1088/1748-9326/abdb5f

[10] Diebold FX, Rudebusch GD, Göbel M, Goulet Coulombe P, Zhang B. When will Arctic sea ice disappear? Projections of area, extent, thickness, and volume. Journal of Econometrics. 2023;236(2):105479. doi: 10.1016/j.jeconom.2023.105479

[11] Zhang J, Spitz YH, Steele M, Ashjian C, Campbell R, Berline L, et al. Modeling the impact of declining sea ice on the Arctic marine planktonic ecosystem. J Geophys Res. 2010;115(C10). doi: 10.1029/2009jc005387

[12] Tremblay J-É, Anderson LG, Matrai P, Coupel P, Bélanger S, Michel C, et al. Global and regional drivers of nutrient supply, primary production and CO2 drawdown in the changing Arctic Ocean. Progress in Oceanography. 2015;139:171–96. doi: 10.1016/j.pocean.2015.08.009

[13] Sejr MK, Poste AE, Renaud PE. Multiple climatic drivers increase pace and consequences of ecosystem change in the Arctic Coastal Ocean. Limnol Oceanogr Letters. 2024;9(6):683–95. doi: 10.1002/lol2.10431

[14] Nummelin A, Ilicak M, Li C, Smedsrud LH. Consequences of future increased Arctic runoff on Arctic Ocean stratification, circulation, and sea ice cover. JGR Oceans. 2016;121(1):617–37. doi: 10.1002/2015jc011156

[15] Harris CM, McTigue ND, McClelland JW, Dunton KH. Do high Arctic coastal food webs rely on a terrestrial carbon subsidy? Food Webs. 2018;15:e00081. doi: 10.1016/j.fooweb.2018.e00081

[16] Tanski G, Wagner D, Knoblauch C, Fritz M, Sachs T, Lantuit H. Rapid CO2 Release From Eroding Permafrost in Seawater. Geophysical Research Letters. 2019;46(20):11244–52. doi: 10.1029/2019gl084303

[17] Terhaar J, Lauerwald R, Regnier P, Gruber N, Bopp L. Around one third of current Arctic Ocean primary production sustained by rivers and coastal erosion. Nat Commun. 2021;12(1):169. doi: 10.1038/s41467-020-20470-z 33420093 PMC7794587

[18] Behnke MI, Tank SE, McClelland JW, Holmes RM, Haghipour N, Eglinton TI, et al. Aquatic biomass is a major source to particulate organic matter export in large Arctic rivers. Proc Natl Acad Sci U S A. 2023;120(12):e2209883120. doi: 10.1073/pnas.2209883120 36913572 PMC10041151

[19] Lannuzel D, Tedesco L, van Leeuwe M, Campbell K, Flores H, Delille B, et al. The future of Arctic sea-ice biogeochemistry and ice-associated ecosystems. Nat Clim Chang. 2020;10(11):983–92. doi: 10.1038/s41558-020-00940-4

[20] Lewis KM, van Dijken GL, Arrigo KR. Changes in phytoplankton concentration now drive increased Arctic Ocean primary production. Science. 2020;369(6500):198–202. doi: 10.1126/science.aay8380 32647002

[21] Bélanger S, Babin M, Tremblay J-É. Increasing cloudiness in Arctic damps the increase in phytoplankton primary production due to sea ice receding. Biogeosciences. 2013;10(6):4087–101. doi: 10.5194/bg-10-4087-2013

[22] Singh RK, Vader A, Mundy CJ, Søreide JE, Iken K, Dunton KH, et al. Satellite-Derived Photosynthetically Available Radiation at the Coastal Arctic Seafloor. Remote Sensing. 2022;14(20):5180. doi: 10.3390/rs14205180

[23] Attard K, Singh RK, Gattuso J-P, Filbee-Dexter K, Krause-Jensen D, Kühl M, et al. Seafloor primary production in a changing Arctic Ocean. Proc Natl Acad Sci U S A. 2024;121(11):e2303366121. doi: 10.1073/pnas.2303366121 38437536 PMC10945780

[24] Boecklen WJ, Yarnes CT, Cook BA, James AC. On the Use of Stable Isotopes in Trophic Ecology. Annu Rev Ecol Evol Syst. 2011;42(1):411–40. doi: 10.1146/annurev-ecolsys-102209-144726

[25] Hutchinson GE. Concluding Remarks. Cold Spring Harbor Symposia on Quantitative Biology. 1957;22(0):415–27. doi: 10.1101/sqb.1957.022.01.039

[26] Layman CA, Arrington DA, Montaña CG, Post DM. Can stable isotope ratios provide for community-wide measures of trophic structure? Ecology. 2007;88(1):42–8. doi: 10.1890/0012-9658(2007)88[42:csirpf]2.0.co;2 17489452

[27] Bearhop S, Adams CE, Waldron S, Fuller RA, Macleod H. Determining trophic niche width: a novel approach using stable isotope analysis. Journal of Animal Ecology. 2004;73: 1007–12. doi: 10.1111/j.0021-8790.2004.00861.x

[28] Newsome SD, Martinez del Rio C, Bearhop S, Phillips DL. A niche for isotopic ecology. Frontiers in Ecology and the Environment. 2007;5(8):429–36. doi: 10.1890/060150.1

[29] Jackson AL, Inger R, Parnell AC, Bearhop S. Comparing isotopic niche widths among and within communities: SIBER - Stable Isotope Bayesian Ellipses in R: Bayesian isotopic niche metrics. J Anim Ecol. 2011;80(3):595–602. doi: 10.1111/j.1365-2656.2011.01806.x 21401589

[30] Swanson HK, Lysy M, Power M, Stasko AD, Johnson JD, Reist JD. A new probabilistic method for quantifying n-dimensional ecological niches and niche overlap. Ecology. 2015;96(2):318–24. doi: 10.1890/14-0235.1 26240852

[31] Cucherousset J, Villéger S. Quantifying the multiple facets of isotopic diversity: New metrics for stable isotope ecology. Ecological Indicators. 2015;56:152–60. doi: 10.1016/j.ecolind.2015.03.032

[32] Iken K, Bluhm B, Dunton K. Benthic food-web structure under differing water mass properties in the southern Chukchi Sea. Deep Sea Research Part II: Topical Studies in Oceanography. 2010;57(1–2):71–85. doi: 10.1016/j.dsr2.2009.08.007

[33] McMahon KW, Hamady LL, Thorrold SR. A review of ecogeochemistry approaches to estimating movements of marine animals. Limnology & Oceanography. 2013;58(2):697–714. doi: 10.4319/lo.2013.58.2.0697

[34] Dunton KH, Weingartner T, Carmack EC. The nearshore western Beaufort Sea ecosystem: Circulation and importance of terrestrial carbon in arctic coastal food webs. Progress in Oceanography. 2006;71(2–4):362–78. doi: 10.1016/j.pocean.2006.09.011

[35] de la Vega C, Jeffreys RM, Tuerena R, Ganeshram R, Mahaffey C. Temporal and spatial trends in marine carbon isotopes in the Arctic Ocean and implications for food web studies. Glob Chang Biol. 2019;25(12):4116–30. doi: 10.1111/gcb.14832 31498935 PMC6899536

[36] Wassmann P, Krause-Jensen D, Bluhm BA, Janout M. Editorial: Towards a Unifying Pan-Arctic Perspective of the Contemporary and Future Arctic Ocean. Front Mar Sci. 2021;8. doi: 10.3389/fmars.2021.678420

[37] Krause-Jensen D, Archambault P, Assis J, Bartsch I, Bischof K, Filbee-Dexter K, et al. Imprint of Climate Change on Pan-Arctic Marine Vegetation. Front Mar Sci. 2020;7. doi: 10.3389/fmars.2020.617324

[38] Ardyna M, Mundy CJ, Mayot N, Matthes LC, Oziel L, Horvat C, et al. Under-Ice Phytoplankton Blooms: Shedding Light on the “Invisible” Part of Arctic Primary Production. Front Mar Sci. 2020;7. doi: 10.3389/fmars.2020.608032

[39] Wiedmann I, Ershova E, Bluhm BA, Nöthig E-M, Gradinger RR, Kosobokova K, et al. What Feeds the Benthos in the Arctic Basins? Assembling a Carbon Budget for the Deep Arctic Ocean. Front Mar Sci. 2020;7. doi: 10.3389/fmars.2020.00224

[40] Jones T, McLennan D, Behe C, Arvnes M, Wesseberg S, Sergienko L, et al. Arctic Coastal Biodiversity Monitoring Plan. Conservation of Arctic Flora and Fauna International Secretariat. Akureyri, Iceland ISBN 978-9935-431-76-9. 2019.

[41] Beaufort Lagoon Ecosystems LTER. Carbon and nitrogen content and stable isotope compositions from biota samples from lagoon sites along the Alaska Beaufort Sea coast, 2018-ongoing. Environmental Data Initiative; 2023. doi: 10.6073/pasta/04a064bbea70a0ec24cad6c1418e57bf

[42] Beaufort Lagoon Ecosystems LTER. Carbon and nitrogen content and stable isotope compositions from particulate organic matter samples from lagoon, river, and open ocean sites along the Alaska Beaufort Sea coast, 2018-ongoing. Environmental Data Initiative; 2023. doi: 10.6073/pasta/9dc939847dfa690d2b2e85457f60436f

[43] Beaufort Lagoon Ecosystems LTER. Carbon and nitrogen content and stable isotope composition from sediment organic matter from lagoon sites along the Alaska Beaufort Sea coast, 2018-ongoing. Environmental Data Initiative; 2024. doi: 10.6073/pasta/207936f4769a6b61a702635718dfa4b0

[44] Bell L, Bluhm B, Iken K. Influence of terrestrial organic matter in marine food webs of the Beaufort Sea shelf and slope. Mar Ecol Prog Ser. 2016;550:1–24. doi: 10.3354/meps11725

[45] Bridier G, Meziane T, Grall J, Chauvaud L, Sejr M, Menneteau S, et al. Coastal waters freshening and extreme seasonality affect organic matter sources, quality, and transfers in a High Arctic fjord (Young Sound, Greenland). Mar Ecol Prog Ser. 2019;610:15–31. doi: 10.3354/meps12857

[46] Bridier G, Olivier F, Chauvaud L, Sejr MK, Grall J. Food source diversity, trophic plasticity, and omnivory enhance the stability of a shallow benthic food web from a high‐Arctic fjord exposed to freshwater inputs. Limnology & Oceanography. 2021;66(S1). doi: 10.1002/lno.11688

[47] De Cesare S, Meziane T, Chauvaud L, Richard J, Sejr M, Thébault J, et al. Dietary plasticity in the bivalve Astarte moerchi revealed by a multimarker study in two Arctic fjords. Mar Ecol Prog Ser. 2017;567:157–72. doi: 10.3354/meps12035

[48] Divine L, Iken K, Bluhm B. Regional benthic food web structure on the Alaska Beaufort Sea shelf. Mar Ecol Prog Ser. 2015;531:15–32. doi: 10.3354/meps11340

[49] Dunton KH, Schonberg SV, Cooper LW. Food Web Structure of the Alaskan Nearshore Shelf and Estuarine Lagoons of the Beaufort Sea. Estuaries and Coasts. 2012;35(2):416–35. doi: 10.1007/s12237-012-9475-1

[50] Ehrman AD, Swanson H, MacPhee S, Majewski A, Archambault P, Eert J, et al. Isotopic and traits-based trophic diversity of Canadian Beaufort Sea benthic communities in relation to food supply. Arctic Science. 2022;8(4):1148–64. doi: 10.1139/as-2021-0040

[51] Gaillard B, Meziane T, Tremblay R, Archambault P, Blicher M, Chauvaud L, et al. Food resources of the bivalve Astarte elliptica in a sub-Arctic fjord: a multi-biomarker approach. Mar Ecol Prog Ser. 2017;567:139–56. doi: 10.3354/meps12036

[52] Gradinger R, Kaufman M, Bluhm B. Pivotal role of sea ice sediments in the seasonal development of near-shore Arctic fast ice biota. Mar Ecol Prog Ser. 2009;394:49–63. doi: 10.3354/meps08320

[53] Gradinger R, Bluhm B. First of an Arctic sea ice meiofauna food web analysis based on abundance, biomass and stable isotope ratios. Mar Ecol Prog Ser. 2020;634:29–43. doi: 10.3354/meps13170

[54] McGovern M, Berge J, Szymczycha B, Weęsławski J, Renaud P. Hyperbenthic food-web structure in an Arctic fjord. Mar Ecol Prog Ser. 2018;603:29–46. doi: 10.3354/meps12713

[55] McGovern M, Poste AE, Oug E, Renaud PE, Trannum HC. Riverine impacts on benthic biodiversity and functional traits: A comparison of two sub-Arctic fjords. Estuarine, Coastal and Shelf Science. 2020;240:106774. doi: 10.1016/j.ecss.2020.106774

[56] McMeans B, Rooney N, Arts M, Fisk A. Food web structure of a coastal Arctic marine ecosystem and implications for stability. Mar Ecol Prog Ser. 2013;482:17–28. doi: 10.3354/meps10278

[57] McTigue ND, Dunton KH. Trophodynamics and organic matter assimilation pathways in the northeast Chukchi Sea, Alaska. Deep Sea Research Part II: Topical Studies in Oceanography. 2014;102:84–96. doi: 10.1016/j.dsr2.2013.07.016

[58] McTigue ND, Dunton KH. Trophodynamics of the Hanna Shoal Ecosystem (Chukchi Sea, Alaska): Connecting multiple end-members to a rich food web. Deep Sea Research Part II: Topical Studies in Oceanography. 2017;144:175–89. doi: 10.1016/j.dsr2.2017.08.010

[59] Renaud PE, Tessmann M, Evenset A, Christensen GN. Benthic food-web structure of an Arctic fjord (Kongsfjorden, Svalbard). Marine Biology Research. 2010;7(1):13–26. doi: 10.1080/17451001003671597

[60] Renaud PE, LÃ¸kken TS, JÃ¸rgensen LL, Berge J, Johnson BJ. Macroalgal detritus and food-web subsidies along an Arctic fjord depth-gradient. Front Mar Sci. 2015;2. doi: 10.3389/fmars.2015.00031

[61] Roy V, Iken K, Gosselin M, Tremblay J-É, Bélanger S, Archambault P. Benthic faunal assimilation pathways and depth-related changes in food-web structure across the Canadian Arctic. Deep Sea Research Part I: Oceanographic Research Papers. 2015;102:55–71. doi: 10.1016/j.dsr.2015.04.009

[62] Søreide JE, Hop H, Carroll ML, Falk-Petersen S, Hegseth EN. Seasonal food web structures and sympagic–pelagic coupling in the European Arctic revealed by stable isotopes and a two-source food web model. Progress in Oceanography. 2006;71(1):59–87. doi: 10.1016/j.pocean.2006.06.001

[63] Søreide JE, Leu E, Berge J, Graeve M, Falk‐Petersen S. Timing of blooms, algal food quality and Calanus glacialis reproduction and growth in a changing Arctic. Global Change Biology. 2010;16(11):3154–63. doi: 10.1111/j.1365-2486.2010.02175.x

[64] Søreide JE, Carroll ML, Hop H, Ambrose WG Jr, Hegseth EN, Falk-Petersen S. Sympagic-pelagic-benthic coupling in Arctic and Atlantic waters around Svalbard revealed by stable isotopic and fatty acid tracers. Marine Biology Research. 2013;9(9):831–50. doi: 10.1080/17451000.2013.775457

[65] Stasko A, Bluhm B, Michel C, Archambault P, Majewski A, Reist J, et al. Benthic-pelagic trophic coupling in an Arctic marine food web along vertical water mass and organic matter gradients. Mar Ecol Prog Ser. 2018;594:1–19. doi: 10.3354/meps12582

[66] Włodarska‐Kowalczuk M, Aune M, Michel LN, Zaborska A, Legeżyńska J. Is the trophic diversity of marine benthic consumers decoupled from taxonomic and functional trait diversity? Isotopic niches of Arctic communities. Limnology & Oceanography. 2019;64(5):2140–51. doi: 10.1002/lno.11174

[67] Zaborska A, Włodarska-Kowalczuk M, Legeżyńska J, Jankowska E, Winogradow A, Deja K. Sedimentary organic matter sources, benthic consumption and burial in west Spitsbergen fjords – Signs of maturing of Arctic fjordic systems? Journal of Marine Systems. 2018;180:112–23. doi: 10.1016/j.jmarsys.2016.11.005

[68] Degen R, Faulwetter S. The Arctic Traits Database – a repository of Arctic benthic invertebrate traits. Earth Syst Sci Data. 2019;11(1):301–22. doi: 10.5194/essd-11-301-2019

[69] Macdonald TA, Burd BJ, Macdonald VI, van Roodselaar A. Taxonomic and Feeding Guild Classification for the Marine Benthic Macroinvertebrates of the Strait of Georgia, British Columbia. 2010.

[70] Post DM, Layman CA, Arrington DA, Takimoto G, Quattrochi J, Montaña CG. Getting to the fat of the matter: models, methods and assumptions for dealing with lipids in stable isotope analyses. Oecologia. 2007;152(1):179–89. doi: 10.1007/s00442-006-0630-x 17225157

[71] Logan JM, Jardine TD, Miller TJ, Bunn SE, Cunjak RA, Lutcavage ME. Lipid corrections in carbon and nitrogen stable isotope analyses: comparison of chemical extraction and modelling methods. J Anim Ecol. 2008;77(4):838–46. doi: 10.1111/j.1365-2656.2008.01394.x 18489570

[72] R Core Team. R: A language and environment for statistical computing. Vienna, Austria; 2022. Available: https://www.R-project.org/

[73] Posit team. RStudio: Integrated Development Environment for R. Boston, MA; 2024. Available: http://www.posit.co/

[74] Muench T, Dolman A, Laepple T. grfxtools: An R package of tools to assist with various plotting tasks. R package version 051, commit bdd5c98e5ae3616c0ddab417cd4e257f3e61881d https://github.com/EarthSystemDiagnostics/grfxtools. 2025.

[75] Lysy M, Asgharian M, Partovi Nia V. A convergence diagnostic for Bayesian clustering. WIREs Computational Stats. 2020;13(4). doi: 10.1002/wics.1536

[76] Mintenbeck K, Jacob U, Knust R, Arntz WE, Brey T. Depth-dependence in stable isotope ratio δ15N of benthic POM consumers: The role of particle dynamics and organism trophic guild. Deep Sea Research Part I: Oceanographic Research Papers. 2007;54(6):1015–23. doi: 10.1016/j.dsr.2007.03.005

[77] Stasko AD, Bluhm BA, Reist JD, Swanson H, Power M. Relationships between depth and δ15N of Arctic benthos vary among regions and trophic functional groups. Deep Sea Research Part I: Oceanographic Research Papers. 2018;135:56–64. doi: 10.1016/j.dsr.2018.03.010

[78] Bacastow RB, Keeling CD, Lueker TJ, Wahlen M, Mook WG. The 13C Suess Effect in the world surface oceans and its implications for oceanic uptake of CO2: Analysis of observations at Bermuda. Global Biogeochemical Cycles. 1996;10(2):335–46. doi: 10.1029/96gb00192

[79] Ko YH, Quay PD. Origin and Accumulation of an Anthropogenic CO2 and 13C Suess Effect in the Arctic Ocean. Global Biogeochemical Cycles. 2020;34(2). doi: 10.1029/2019gb006423

[80] Gradinger R. Sea-ice algae: Major contributors to primary production and algal biomass in the Chukchi and Beaufort Seas during May/June 2002. Deep Sea Research Part II: Topical Studies in Oceanography. 2009;56(17):1201–12. doi: 10.1016/j.dsr2.2008.10.016

[81] Humborg C, Geibel MC, Anderson LG, Björk G, Mörth C, Sundbom M, et al. Sea‐air exchange patterns along the central and outer East Siberian Arctic Shelf as inferred from continuous CO2, stable isotope, and bulk chemistry measurements. Global Biogeochemical Cycles. 2017;31(7):1173–91. doi: 10.1002/2017gb005656

[82] Grebmeier JM, Cooper LW, Feder HM, Sirenko BI. Ecosystem dynamics of the Pacific-influenced Northern Bering and Chukchi Seas in the Amerasian Arctic. Progress in Oceanography. 2006;71(2–4):331–61. doi: 10.1016/j.pocean.2006.10.001

[83] Cautain IJ, Last KS, Bluhm BA, Renaud PE, McKee D, Narayanaswamy BE. High uptake of sympagic organic matter by benthos on an Arctic outflow shelf. PLoS One. 2024;19(8):e0308562. doi: 10.1371/journal.pone.0308562 39110718 PMC11305566

[84] Niemi A, Bluhm BA, Juul-Pedersen T, Kohlbach D, Reigstad M, Søgaard DH, et al. Ice algae contributions to the benthos during a time of sea ice change: a review of supply, coupling, and fate. Front Environ Sci. 2024;12. doi: 10.3389/fenvs.2024.1432761

[85] McTigue ND, Bucolo P, Liu Z, Dunton KH. Pelagic-benthic coupling, food webs, and organic matter degradation in the Chukchi Sea: Insights from sedimentary pigments and stable carbon isotopes: Chukchi Sea sedimentary pigments. Limnol Oceanogr. 2015;60(2):429–45. doi: 10.1002/lno.10038

[86] Dunton KH, Goodall JL, Schonberg SV, Grebmeier JM, Maidment DR. Multi-decadal synthesis of benthic–pelagic coupling in the western arctic: Role of cross-shelf advective processes. Deep Sea Research Part II: Topical Studies in Oceanography. 2005;52(24–26):3462–77. doi: 10.1016/j.dsr2.2005.09.007

[87] Fripiat F, Declercq M, Sapart CJ, Anderson LG, Bruechert V, Deman F, et al. Influence of the bordering shelves on nutrient distribution in the Arctic halocline inferred from water column nitrate isotopes. Limnology & Oceanography. 2018;63(5):2154–70. doi: 10.1002/lno.10930

[88] Mincks S, Smith C, DeMaster D. Persistence of labile organic matter and microbial biomass in Antarctic shelf sediments: evidence of a sediment “food bank”. Marine Ecology Progress Series. 2005;300: 3–19. doi: 10.3354/meps300003

[89] Ye L, Yu X, Liu Y, Astakhov AS, Bosin A, Bian Y, et al. Organic carbon burial dynamics at the Chukchi Shelf margin: Implications for the Arctic Ocean carbon sink. Palaeogeography, Palaeoclimatology, Palaeoecology. 2024;655:112534. doi: 10.1016/j.palaeo.2024.112534

[90] Belicka LL, Harvey HR. The sequestration of terrestrial organic carbon in Arctic Ocean sediments: A comparison of methods and implications for regional carbon budgets. Geochimica et Cosmochimica Acta. 2009;73(20):6231–48. doi: 10.1016/j.gca.2009.07.020

[91] Schreiner KM, Bianchi TS, Eglinton TI, Allison MA, Hanna AJM. Sources of terrigenous inputs to surface sediments of the Colville River Delta and Simpson’s Lagoon, Beaufort Sea, Alaska: INPUTS TO THE COLVILLE RIVER DELTA, AK. JGR Biogeosciences. 2013;118(2):808–24. doi: 10.1002/jgrg.20065

[92] McMahon K, Ambrose WG J, Johnson B, Sun M, Lopez G, Clough L, et al. Benthic community response to ice algae and phytoplankton in Ny Ålesund, Svalbard. Mar Ecol Prog Ser. 2006;310:1–14. doi: 10.3354/meps310001

[93] McMahon KW, Ambrose WG Jr, Reynolds MJ, Johnson BJ, Whiting A, Clough LM. Arctic lagoon and nearshore food webs: Relative contributions of terrestrial organic matter, phytoplankton, and phytobenthos vary with consumer foraging dynamics. Estuarine, Coastal and Shelf Science. 2021;257:107388. doi: 10.1016/j.ecss.2021.107388

[94] Zinkann A-C, Wooller MJ, O’Brien D, Iken K. Does feeding type matter? Contribution of organic matter sources to benthic invertebrates on the Arctic Chukchi Sea shelf. Food Webs. 2021;29:e00205. doi: 10.1016/j.fooweb.2021.e00205

[95] Yunda-Guarin G, Michel L, Nozais C, Archambault P. Interspecific differences in feeding selectivity shape isotopic niche structure of three ophiuroids in the Arctic Ocean. Mar Ecol Prog Ser. 2022;683:81–95. doi: 10.3354/meps13965

[96] Yunda-Guarin G, Michel LN, Roy V, Friscourt N, Gosselin M, Nozais C, et al. Trophic ecology of epibenthic communities exposed to different sea-ice concentrations across the Canadian Arctic Ocean. Progress in Oceanography. 2023;217:103105. doi: 10.1016/j.pocean.2023.103105

[97] Macdonald RW, Naidu AS, Yunker MB, Gobeil C. The Beaufort Sea: Distribution, sources, fluxes, and burial rates of organic carbon. In: Stein R, Macdonald RW, editors. The Organic Carbon Cycle in the Arctic Ocean. Berlin Hedelberg, Germany: Springer; 2004. p. 177–93.

[98] Pickart RS. Shelfbreak circulation in the Alaskan Beaufort Sea: Mean structure and variability. J Geophys Res. 2004;109(C4). doi: 10.1029/2003jc001912

[99] Stanek AE, von Biela VR, Laske SM, Taylor RL, Dunton KH. Barrier islands influence the assimilation of terrestrial energy in nearshore fishes. Estuarine, Coastal and Shelf Science. 2022;278:108094. doi: 10.1016/j.ecss.2022.108094

[100] Rafter PA, Bagnell A, Marconi D, DeVries T. Global trends in marine nitrate N isotopes from observations and a neural network-based climatology. Biogeosciences. 2019;16(13):2617–33. doi: 10.5194/bg-16-2617-2019

[101] Polyakov IV, Pnyushkov AV, Alkire MB, Ashik IM, Baumann TM, Carmack EC, et al. Greater role for Atlantic inflows on sea-ice loss in the Eurasian Basin of the Arctic Ocean. Science. 2017;356(6335):285–91. doi: 10.1126/science.aai8204 28386025

[102] Yu X, Ye L, Bian Y, Liu Y, Zhang W, Wang W, et al. Composition and sources of suspended particles in the Pacific Arctic region. Mar Environ Res. 2023;191:106127. doi: 10.1016/j.marenvres.2023.106127 37573738

[103] Wilce RT, Dunton KH. The Boulder Patch (North Alaska, Beaufort Sea) and its Benthic Algal Flora. Arctic. 2014;67: 43–56.

[104] Dunton KH, Schell DM. Dependence of consumers on macroalgal (Laminaria solidungula) carbon in an arctic kelp community: ?13C evidence. Mar Biol. 1987;93(4):615–25. doi: 10.1007/bf00392799

[105] Duncan RJ, Søreide JE, Varpe Ø, Wiktor J, Pitusi V, Runge E, et al. Spatio-temporal dynamics in microalgal communities in Arctic land-fast sea ice. Progress in Oceanography. 2024;224:103248. doi: 10.1016/j.pocean.2024.103248

[106] Pineault S, Tremblay J, Gosselin M, Thomas H, Shadwick E. The isotopic signature of particulate organic C and N in bottom ice: Key influencing factors and applications for tracing the fate of ice‐algae in the Arctic Ocean. JGR Oceans. 2013;118(1):287–300. doi: 10.1029/2012jc008331

[107] Tremblay J-É, Michel C, Hobson KA, Gosselin M, Price NM. Bloom dynamics in early opening waters of the Arctic Ocean. Limnol Oceanogr. 2006;51(2):900–12. doi: 10.4319/lo.2006.51.2.0900

[108] Grippo MA, Fleeger JW, Dubois SF, Condrey R. Spatial variation in basal resources supporting benthic food webs revealed for the inner continental shelf. Limnology & Oceanography. 2011;56(3):841–56. doi: 10.4319/lo.2011.56.3.0841

[109] Kang C-K, Park HJ, Choy EJ, Choi K-S, Hwang K, Kim J-B. Linking Intertidal and Subtidal Food Webs: Consumer-Mediated Transport of Intertidal Benthic Microalgal Carbon. PLoS One. 2015;10(10):e0139802. doi: 10.1371/journal.pone.0139802 26448137 PMC4598165

[110] Oxtoby LE, Mathis JT, Juranek LW, Wooller MJ. Estimating stable carbon isotope values of microphytobenthos in the Arctic for application to food web studies. Polar Biol. 2015;39(3):473–83. doi: 10.1007/s00300-015-1800-2

[111] Pasotti F, Saravia LA, De Troch M, Tarantelli MS, Sahade R, Vanreusel A. Benthic Trophic Interactions in an Antarctic Shallow Water Ecosystem Affected by Recent Glacier Retreat. PLoS One. 2015;10(11):e0141742. doi: 10.1371/journal.pone.0141742 26559062 PMC4641631

[112] Wulff A, Iken K, Quartino ML, Al-Handal A, Wiencke C, Clayton MN. Biodiversity, biogeography and zonation of marine benthic micro- and macroalgae in the Arctic and Antarctic. botm. 2009;52(6):491–507. doi: 10.1515/bot.2009.072

[113] Leu E, Søreide JE, Hessen DO, Falk-Petersen S, Berge J. Consequences of changing sea-ice cover for primary and secondary producers in the European Arctic shelf seas: Timing, quantity, and quality. Progress in Oceanography. 2011;90(1–4):18–32. doi: 10.1016/j.pocean.2011.02.004

[114] Schollmeier T, Oliveira ACM, Wooller MJ, Iken K. Tracing sea ice algae into various benthic feeding types on the Chukchi Sea shelf. Polar Biol. 2017;41(2):207–24. doi: 10.1007/s00300-017-2182-4

[115] Koch CW, Brown TA, Amiraux R, Ruiz-Gonzalez C, MacCorquodale M, Yunda-Guarin GA, et al. Year-round utilization of sea ice-associated carbon in Arctic ecosystems. Nat Commun. 2023;14(1):1964. doi: 10.1038/s41467-023-37612-8 37029106 PMC10081986

[116] Fischer G. Stable carbon isotope ratios of plankton carbon and sinking organic matter from the Atlantic sector of the Southern Ocean. Marine Chemistry. 1991;35(1–4):581–96. doi: 10.1016/s0304-4203(09)90044-5

[117] Schubert CJ, Calvert SE. Nitrogen and carbon isotopic composition of marine and terrestrial organic matter in Arctic Ocean sediments: implications for nutrient utilization and organic matter composition. Deep Sea Research Part I: Oceanographic Research Papers. 2001;48:789–810. doi: 10.1016/S0967-0637(00)00069-8

[118] Fredriksen S, Karsten U, Bartsch I, Woelfel J, Koblowsky M, Schumann R, et al. Biodiversity of Benthic Macro- and Microalgae from Svalbard with Special Focus on Kongsfjorden. In: Hop H, Wiencke C, editors. The Ecosystem of Kongsfjorden, Svalbard. Cham: Springer International Publishing; 2019. p. 331–71. doi: 10.1007/978-3-319-46425-1_9

[119] Glud RN, Woelfel J, Karsten U, Kühl M, Rysgaard S. Benthic microalgal production in the Arctic: applied methods and status of the current database. botm. 2009;52(6):559–71. doi: 10.1515/bot.2009.074

[120] Glud R, Kühl M, Wenzhöfer F, Rysgaard S. Benthic diatoms of a high Arctic fjord (Young Sound, NE Greenland): importance for ecosystem primary production. Mar Ecol Prog Ser. 2002;238:15–29. doi: 10.3354/meps238015

[121] Matheke GEM, Horner R. Primary Productivity of the Benthic Microalgae in the Chukchi Sea near Barrow, Alaska. J Fish Res Bd Can. 1974;31(11):1779–86. doi: 10.1139/f74-226

[122] Woelfel J, Schumann R, Peine F, Flohr A, Kruss A, Tegowski J, et al. Microphytobenthos of Arctic Kongsfjorden (Svalbard, Norway): biomass and potential primary production along the shore line. Polar Biol. 2010;33(9):1239–53. doi: 10.1007/s00300-010-0813-0

[123] Dodds WK, Collins SM, Hamilton SK, Tank JL, Johnson S, Webster JR, et al. You are not always what we think you eat: selective assimilation across multiple whole‐stream isotopic tracer studies. Ecology. 2014;95(10):2757–67. doi: 10.1890/13-2276.1

[124] Doi H, Kikuchi E, Takagi S, Shikano S. Selective assimilation by deposit feeders: Experimental evidence using stable isotope ratios. Basic and Applied Ecology. 2006;7(2):159–66. doi: 10.1016/j.baae.2005.04.011

[125] Robbins CT, Felicetti LA, Florin ST. The impact of protein quality on stable nitrogen isotope ratio discrimination and assimilated diet estimation. Oecologia. 2010;162(3):571–9. doi: 10.1007/s00442-009-1485-8 19898979

[126] McCutchan JH Jr, Lewis WM Jr, Kendall C, McGrath CC. Variation in trophic shift for stable isotope ratios of carbon, nitrogen, and sulfur. Oikos. 2003;102(2):378–90. doi: 10.1034/j.1600-0706.2003.12098.x

[127] Nadon M-O, Himmelman JH. Stable isotopes in subtidal food webs: Have enriched carbon ratios in benthic consumers been misinterpreted? Limnology & Oceanography. 2006;51(6):2828–36. doi: 10.4319/lo.2006.51.6.2828

[128] Kaufman MR, Gradinger RR, Bluhm BA, O’Brien DM. Using stable isotopes to assess carbon and nitrogen turnover in the Arctic sympagic amphipod Onisimus litoralis. Oecologia. 2008;158(1):11–22. doi: 10.1007/s00442-008-1122-y 18709389

[129] Weems J, Iken K, Gradinger R, Wooller MJ. Carbon and nitrogen assimilation in the Bering Sea clams Nuculana radiata and Macoma moesta. Journal of Experimental Marine Biology and Ecology. 2012;430–431:32–42. doi: 10.1016/j.jembe.2012.06.015

[130] Barton MB, Litvin SY, Vollenweider JJ, Heintz RA, Norcross BL, Boswell KM. Experimental determination of tissue turnover rates and trophic discrimination factors for stable carbon and nitrogen isotopes of Arctic Sculpin (Myoxocephalus scorpioides): A common Arctic nearshore fish. Journal of Experimental Marine Biology and Ecology. 2019;511:60–7. doi: 10.1016/j.jembe.2018.11.005

[131] Lovvorn J, Cooper L, Brooks M, De Ruyck C, Bump J, Grebmeier J. Organic matter pathways to zooplankton and benthos under pack ice in late winter and open water in late summer in the north-central Bering Sea. Mar Ecol Prog Ser. 2005;291:135–50. doi: 10.3354/meps291135

[132] North CA, Lovvorn JR, Kolts JM, Brooks ML, Cooper LW, Grebmeier JM. Deposit‐feeder diets in the Bering Sea: potential effects of climatic loss of sea ice‐related microalgal blooms. Ecological Applications. 2014;24(6):1525–42. doi: 10.1890/13-0486.1 29160671

[133] Kędra M, Cooper LW, Silberberger MJ, Zhang M, Biasatti D, Grebmeier JM. Organic carbon source variability in Arctic bivalves as deduced from the compound specific carbon isotopic composition of amino acids. Journal of Marine Systems. 2021;219:103547. doi: 10.1016/j.jmarsys.2021.103547

[134] Huxel GR, McCann K, Polis GA. Effects of partitioning allochthonous and autochthonous resources on food web stability. Ecological Research. 2002;17(4):419–32. doi: 10.1046/j.1440-1703.2002.00501.x

[135] McMeans BC, McCann KS, Humphries M, Rooney N, Fisk AT. Food Web Structure in Temporally-Forced Ecosystems. Trends Ecol Evol. 2015;30(11):662–72. doi: 10.1016/j.tree.2015.09.001 26452520

