# Supplementary figures and images for: Trophic niche variation across the pan-Arctic coastal continuum
Source: PLoS One. 2025 Nov 4;20(11):e0335406. doi: 10.1371/journal.pone.0335406 (PMC12585089; doi:10.1371/journal.pone.0335406)

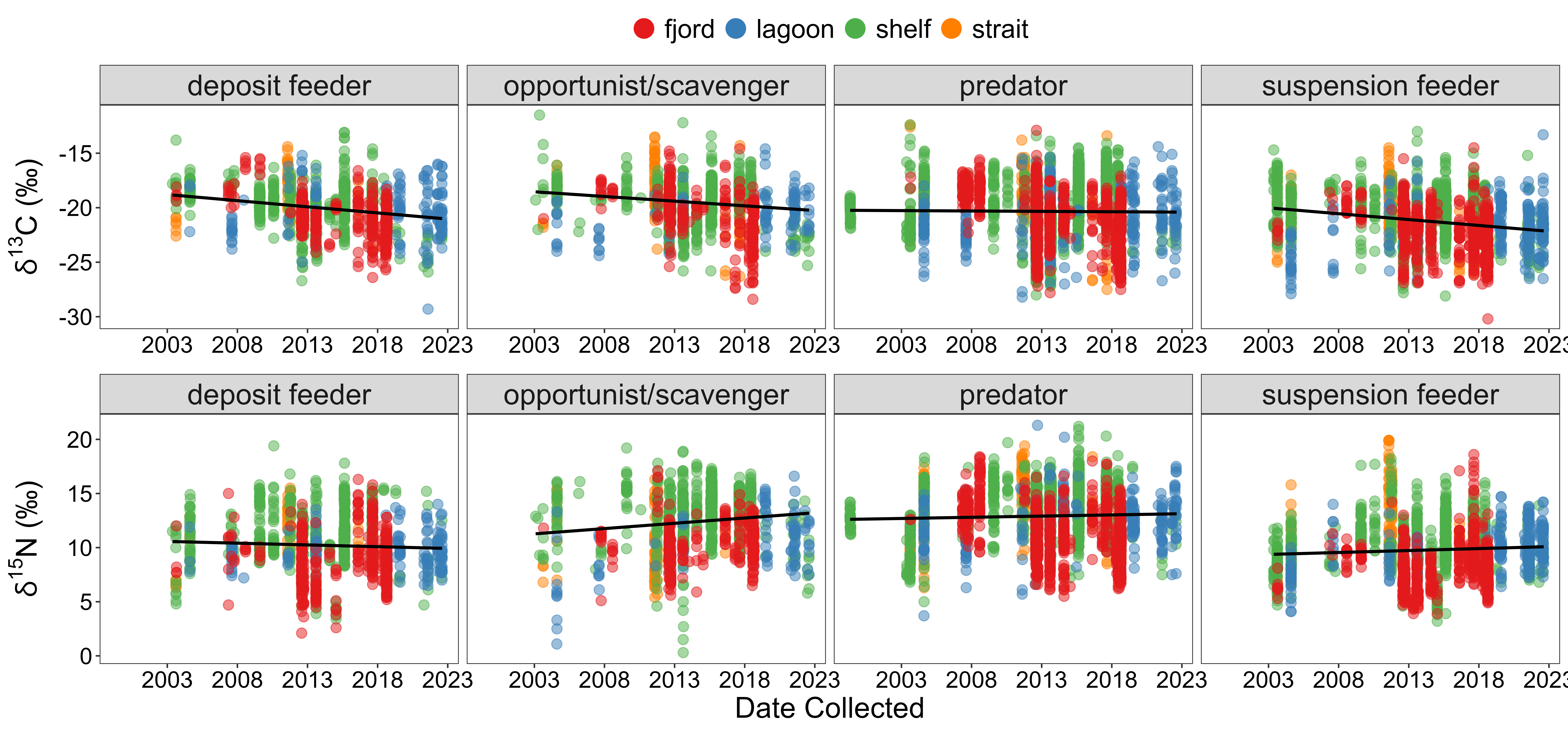

Supplement: S1 Fig — Graphical display of regressions faceted by feeding habit and stable isotope for the linear regressions presented in Table 4. (PNG) [file pone.0335406.s001.png]

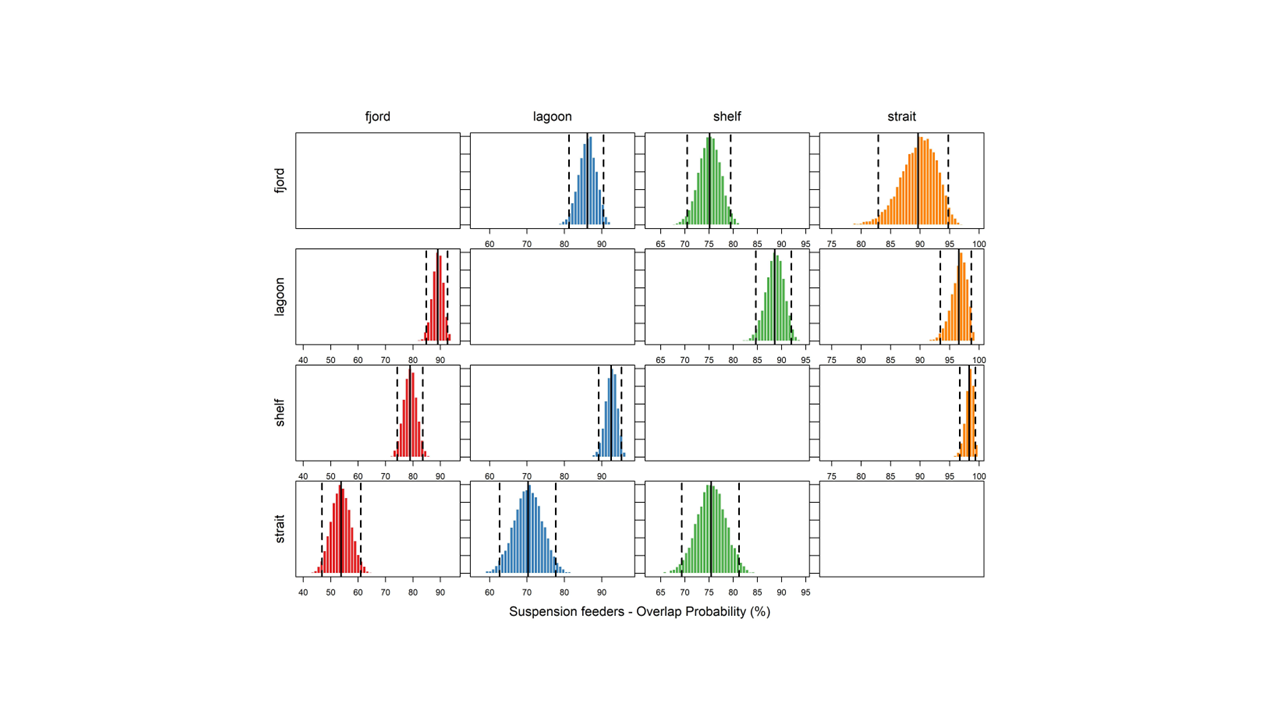

Supplement: S2 Fig — Probability that an individual sampled in the coastscape listed for the row would also occur within the isotopic niche of the same feeding guild samples in the coastscape listed for the column. (TIF) [file pone.0335406.s002.TIF]

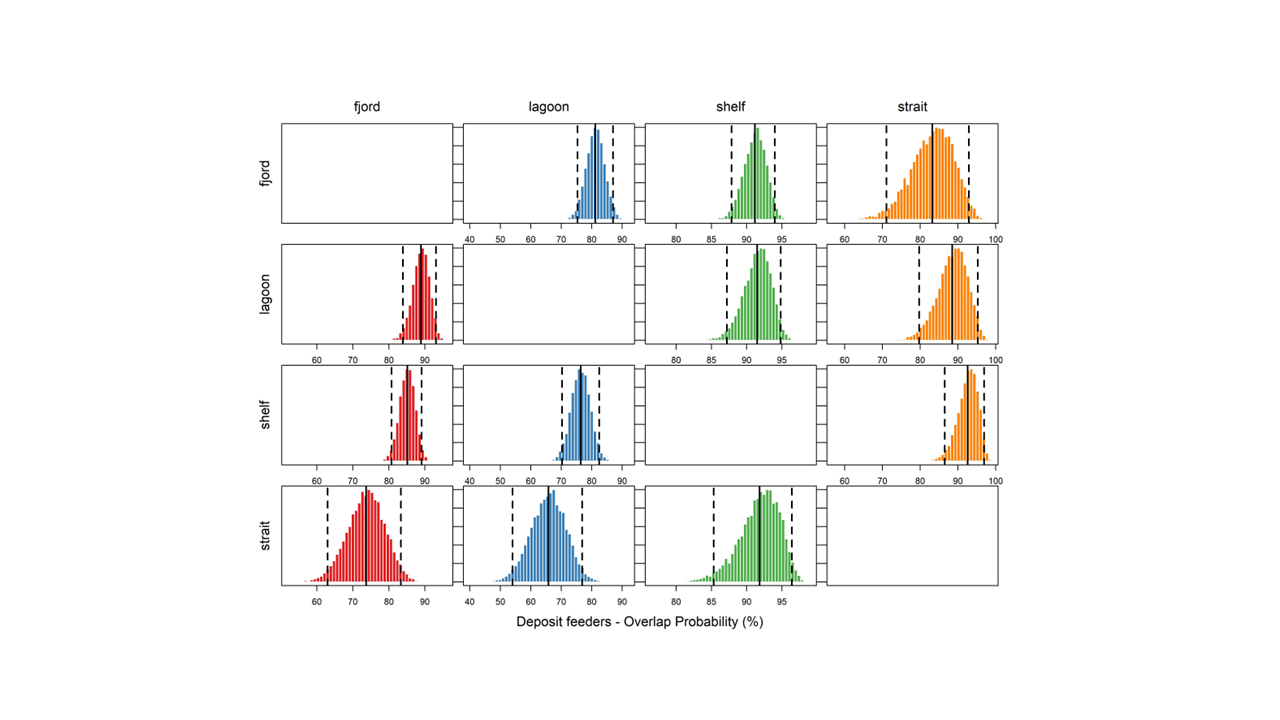

Supplement: S3 Fig — Probability that an individual sampled in the coastscape listed for the row would also occur within the isotopic niche of the same feeding guild samples in the coastscape listed for the column. (TIF) [file pone.0335406.s003.TIF]

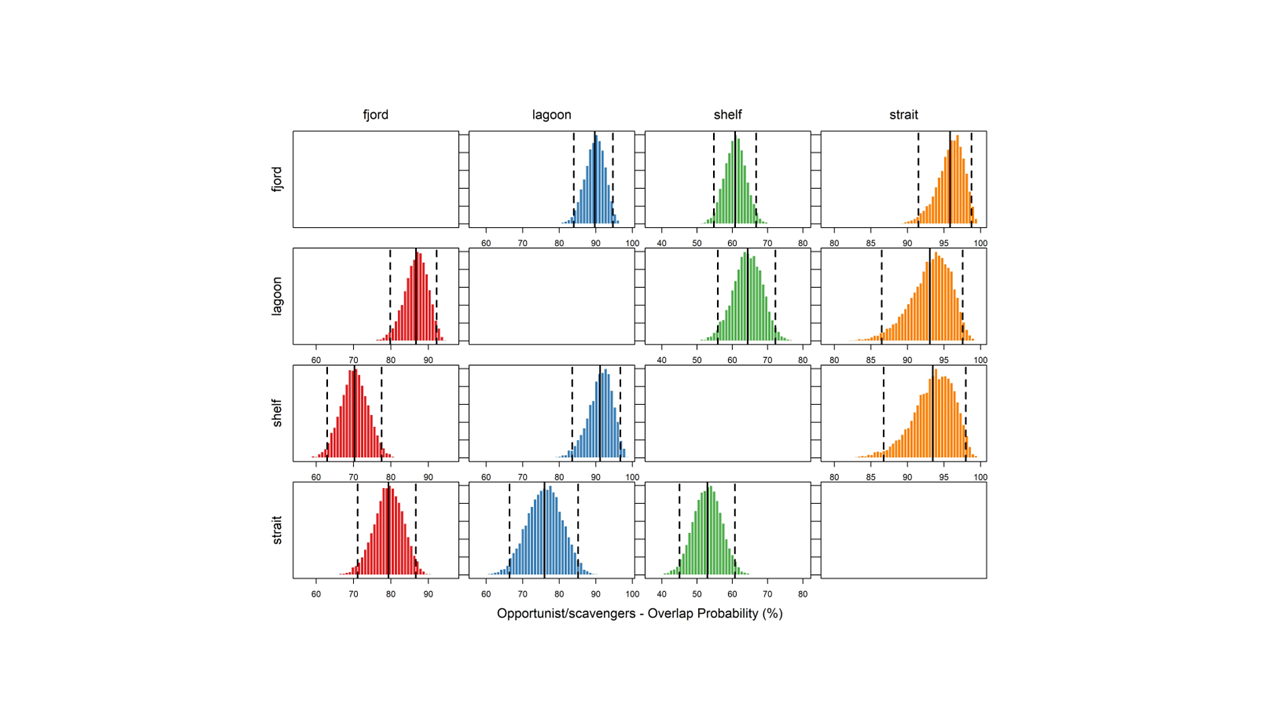

Supplement: S4 Fig — Probability that an individual sampled in the coastscape listed for the row would also occur within the isotopic niche of the same feeding guild samples in the coastscape listed for the column. (TIF) [file pone.0335406.s004.TIF]

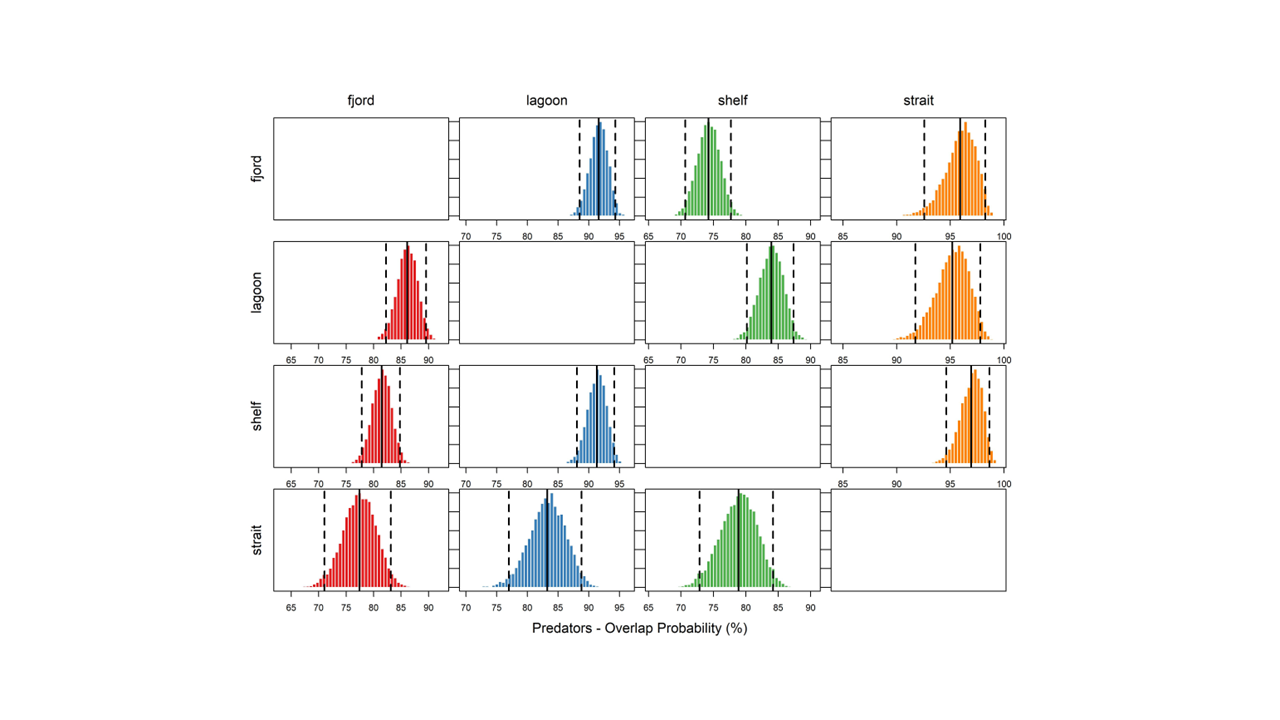

Supplement: S5 Fig — Probability that an individual sampled in the coastscape listed for the row would also occur within the isotopic niche of the same feeding guild samples in the coastscape listed for the column. (TIF) [file pone.0335406.s005.TIF]

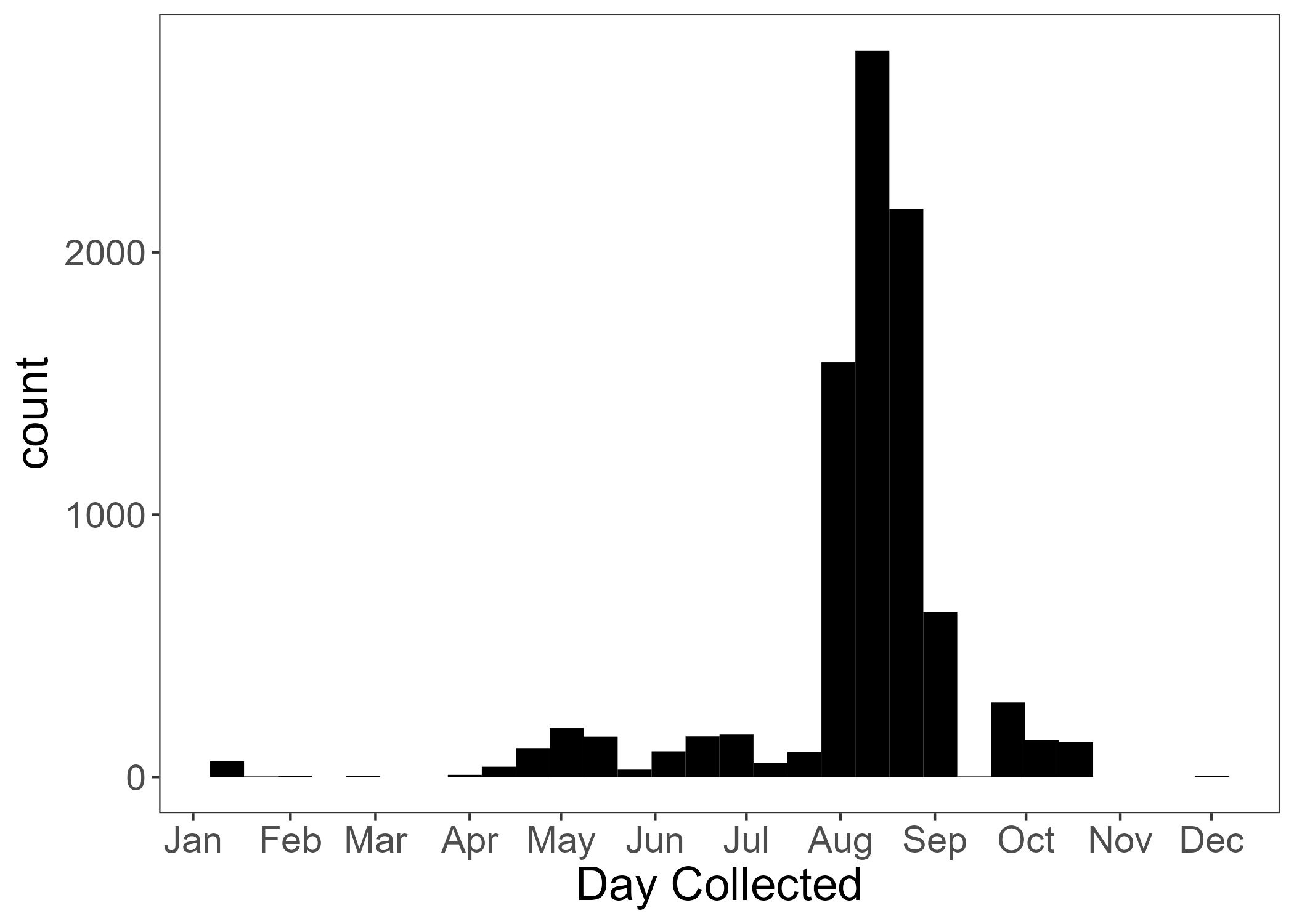

Supplement: S6 Fig — Counts represent rows of data. End-members and consumers from all years included. (PNG) [file pone.0335406.s006.png]
