# Supplementary material for: Trophic niche variation across the pan-Arctic coastal continuum
Source: PLoS One. 2025 Nov 4;20(11):e0335406. doi: 10.1371/journal.pone.0335406 (PMC12585089; doi:10.1371/journal.pone.0335406)
Supplement: S2 Table — Corrections were from equations represented in Table 2. (DOCX) [file pone.0335406.s008.docx]

**S2 Table. Genera in the dataset that were mathematically corrected for lipid content.** Mathematical corrections described in Table 2.

| **Phylum** | **Genus** |
| --- | --- |
| Annelida | Bylgides |
| Arthropoda | Diastylis |
| Arthropoda | Tecticeps |
| Arthropoda | Anonyx |
| Arthropoda | Calanus |
| Arthropoda | Hyas |
| Arthropoda | Argis |
| Arthropoda | Eualus |
| Arthropoda | Lebbeus |
| Arthropoda | Sclerocrangon |
| Arthropoda | Pagurus |
| Arthropoda | Labidochirus |
| Arthropoda | Chionoecetes |
| Arthropoda | Pandalus |
| Bryozoa | Alcyonidium |
| Bryozoa | Eucratea |
| Chordata | Boltenia |
| Chordata | Styela |
| Chordata | Chelyosoma |
| Chordata | Pelonaia |
| Chordata | Ascidia |
| Chordata | Halocynthia |
| Cnidaria | Gersemia |
| Cnidaria | Stomphia |
| Echinodermata | Ophiura |
| Echinodermata | Ocnus |
| Echinodermata | Stegophiura |
| Echinodermata | Leptasterias |
| Echinodermata | Gorgonocephalus |
| Echinodermata | Myriotrochus |
| Echinodermata | Strongylocentrotus |
| Echinodermata | Pteraster |
| Echinodermata | Asterias |
| Echinodermata | Crossaster |
| Echinodermata | Psolus |
| Echinodermata | Echinarachnius |
| Echinodermata | Solaster |
| Mollusca | Tritonia |
| Mollusca | Margarites |
| Mollusca | Tachyrhynchus |
| Mollusca | Amicula |
| Mollusca | Chlamys |
| Mollusca | Cryptonatica |
| Mollusca | Buccinum |
| Mollusca | Musculus |
| Mollusca | Euspira |
| Mollusca | Admete |
| Mollusca | Neptunea |
| Mollusca | Latisipho |
| Porifera | Polymastia |
| Porifera | Halichondria |
| Porifera | Haliclona |
| Porifera | Semisuberites |
