# Supplementary material for: Trophic niche variation across the pan-Arctic coastal continuum
Source: PLoS One. 2025 Nov 4;20(11):e0335406. doi: 10.1371/journal.pone.0335406 (PMC12585089; doi:10.1371/journal.pone.0335406)
Supplement: S1 Table — Full bibliographic information available for each publication listed in References. Projects listed with acronyms represent datasets unpublished in peer-review literature. All data are available in public repository referenced in manuscript. (DOCX) [file pone.0335406.s007.docx]

**S1 Table. Aggregated data sources.** Full bibliographic information available for each publication listed in References. Projects listed with acronyms represent datasets unpublished in peer-review literature. All data are available in public repository referenced in manuscript.

| **Sector** | **Coastscape** | **Source** |
| --- | --- | --- |
| Baffin Bay | fjord | Gaillard et al., 2017; McMeans et al., 2013; Roy et al., 2015 |
| Baffin Bay | shelf | Roy et al., 2015 |
| Beaufort | lagoon | Harris et al., 2018; BLE LTER 2023; Dunton et al., 2012; Gradinger & Bluhm 2020 |
| Beaufort | shelf | Harris et al., 2018; Gradinger & Bluhm 2020; Roy et al., 2015; Bell et al., 2016; BLE LTER 2023; Arctic Nearshore Impact Monitoring in Development Area (ANIMIDA III); Beaufort Regional Environmental Assessment Marine Fishes Project (BREA MFP) |
| Bering/Chukchi | shelf | McTigue & Dunton, 2014; McTigue & Dunton, 2017; Iken et al., 2010; Arctic Marine Biodiversity Observing Network (AMBON); Arctic Shelf Growth, Advection, Respiration and Deposition (ASGARD) |
| Bering/Chukchi | strait | Iken et al., 2010 |
| Canadian Archipelago | fjord | Kitikmeot Sea Science Study (K3S) |
| Canadian Archipelago | shelf | Beaufort Regional Environmental Assessment Marine Fishes Project (BREA MFP) |
| Canadian Archipelago | strait | Roy et al., 2015; Kitikmeot Sea Science Study (K3S) |
| E. Greenland | fjord | DeCesare et al., 2017; Bridier et al., 2019; Bridier et al., 2021 |
| Svalbard | fjord | DeCesare et al., 2017; Renaud et al., 2011; Renaud et al., 2015; Zaborska et al. 2018; Soreide et al., 2013; Włodarska-Kowalczuk et al. 2019; Glaciers as Arctic Ecosystem Refugia (GLAERE); Mare Incognitum (MARINE NIGHT); Effects of TERRestrial inputs on Arctic Coastal Ecosystems (TerrACE) |
| Svalbard | lagoon | Effects of TERRestrial inputs on Arctic Coastal Ecosystems (TerrACE) |
| Svalbard | shelf | Soreide et al., 2006; Soreide et al., 2013; Renaud et al., 2011; Włodarska-Kowalczuk et al. 2019; Mare Incognitum (MARINE NIGHT); |
