# Supplementary material for: Trophic niche variation across the pan-Arctic coastal continuum
Source: PLoS One. 2025 Nov 4;20(11):e0335406. doi: 10.1371/journal.pone.0335406 (PMC12585089; doi:10.1371/journal.pone.0335406)
Supplement: Attachment — Submitted filename: Comments.docx [file pone.0335406.s009.docx]

This manuscript focuses on a highly relevant and ecologically important question: how trophic niche structures in pan-Arctic coastal ecosystems have changed under the dual influence of environmental change and coastal geomorphology. The topic fits well with current global research on Arctic carbon cycling and land–ocean interactions and has clear scientific value. The study compiles and analyses more than 10,000 stable isotope records across multiple trophic levels over the past two decades, showing its strength in data synthesis and regional pattern identification. A particularly commendable contribution is the development and use of the “coastscape” concept, which classifies Arctic coastal systems based on geomorphology, ecological function, and biogeochemical traits. This framework enables meaningful comparisons of consumer isotopic niches across fjords, lagoons, shelves, and straits. The study is a large undertaking with wide spatial and temporal coverage, and it uses appropriate methods such as non-parametric statistics and Bayesian niche overlap modelling (nicheROVER).

However, although the authors demonstrate solid academic ability in building the dataset and analytical framework, some key ecological processes are not discussed in sufficient depth. Generally, the paper does not fully explore the selective assimilation of organic matter by consumers, the role of remineralisation in explaining δ¹⁵N differences in sPOM, or the full pathway of isotopic signal transfer from source to sediment. Also, the assumed δ¹³C values for certain end-members, such as phytoplankton, are somewhat conservative and may lead to misjudging their contribution to consumers. The overall writing is generally clear, but some mechanism discussions lack depth in terms of biogeochemical processes. In summary, I recommend minor revision, as the conclusions are mostly supported by the data and the manuscript is suitable for publication in PLOS ONE after revisions.

**Specific comments:**

**Line 48:** Please shorten the abstract slightly. Focus on the main message and avoid separating the conclusions.

**Lines 75–77:** The phrase “situated in the crucible of climate change” is too rhetorical. Replace it with a more neutral and scientific expression. Similar long or redundant sentences appear elsewhere and should be trimmed for clarity.

**Lines 143–146:** This is a key sentence. Please highlight the novel aspects of this study, especially regarding trophic niche expression and ecological plasticity.

**Line 210:** The description of iPOM should be moved to here from Line 213 for better logical flow.

**Lines 308–311:** Please explain more clearly how “overlap probability” is calculated. A short mathematical description and interpretation would be helpful.

**Line 359:** Although regression results for δ¹³C and δ¹⁵N are reported as significant, model fit indicators (e.g. R² or confidence intervals for Kendall’s tau) should be added in Fig. 2 and the text to strengthen the interpretation.

**Lines 490–493:** Consider adding a rough estimate of freshwater aquatic microalgae input. Based on radiocarbon data, most POM from rivers may be degraded before reaching the coastal ocean (e.g. Goñi et al., 2013).

**Lines 504–507:** The explanation provided is questionable. In the Bering Strait and Chukchi Shelf, terrestrial OM is often more likely to remain suspended in surface waters than marine OM (e.g. Ye et al., 2024). The general statement that terrestrial OM preferentially enters sediments should be reconsidered in light of regional variability. Also, the δ¹⁵N difference between sPOM and pPOM should not be attributed solely to end-member differences. Please consider the influence of mineralisation in surface sediments, including nitrogen regeneration and microbial reworking, and explain why δ¹⁵N is more affected than δ¹³C.

**Lines 513–522:** The manuscript connects isotope values of end-members and consumers over time, but does not clearly describe a full mechanism for signal transfer from pPOM → consumer → sPOM. Please outline the steps of fractionation, assimilation, unassimilated residue, and sedimentation more clearly to strengthen the logic.

**Line 598:** Please recheck the δ¹³C range for Arctic coastal phytoplankton (−26‰ to −23‰), as this may be too low. Several studies report values of about −21‰ or higher in shallow productive waters (e.g. Søreide et al., 2006), and underestimating this range may lead to underestimating phytoplankton contributions.

**Lines 599–601:** Please discuss the possibility that consumers preferentially assimilate ¹³C-rich compounds (e.g. proteins), which could cause consumer δ¹³C values to increase while leaving behind ¹³C-depleted lipids in the remaining pPOM.
