# Supplementary material for: Trophic niche variation across the pan-Arctic coastal continuum
Source: PLoS One. 2025 Nov 4;20(11):e0335406. doi: 10.1371/journal.pone.0335406 (PMC12585089; doi:10.1371/journal.pone.0335406)
Supplement: Attachment — Submitted filename: Reviewer Comments of McTigue et al. PLOS ONE_final.docx [file pone.0335406.s011.docx]

Dear Vitor Hugo Rodrigues Paiva, Liming Ye, and the anonymous reviewer,

We thank you for your feedback and constructive comments that improved this manuscript. We feel the peer-review process has benefited us scientifically and improved the merits of this manuscript for publication. Below, we respond to each comment, both from the handling editor and the two reviewers. Our responses are in **bold and blue** text to facilitate reading the original comments (black typeface) and our responses. We also list the new line number(s) that changes were made when the comment required us to revise the text.

Best regards,

Nathan McTigue

Journal Requirements:

When submitting your revision, we need you to address these additional requirements.

1. Please ensure that your manuscript meets PLOS ONE's style requirements, including those for file naming. The PLOS ONE style templates can be found at

<https://journals.plos.org/plosone/s/file?id=wjVg/PLOSOne_formatting_sample_main_body.pdf> and

<https://journals.plos.org/plosone/s/file?id=ba62/PLOSOne_formatting_sample_title_authors_affiliations.pdf>

**The style requirements have been checked. References have been checked for proper placement of special characters and sub/superscripting.**

2. Please note that PLOS ONE has specific guidelines on code sharing for submissions in which author-generated code underpins the findings in the manuscript. In these cases, we expect all author-generated code to be made available without restrictions upon publication of the work. Please review our guidelines at <https://journals.plos.org/plosone/s/materials-and-software-sharing#loc-sharing-code> and ensure that your code is shared in a way that follows best practice and facilitates reproducibility and reuse.

**No author-generated code underpins the findings in this manuscript.**

3. Thank you for stating the following financial disclosure: [This research was funded through the 2017-2018 Belmont Forum and BiodivERsA joint call for research proposals, under the BiodivScen ERA-Net COFUND program, and with the funding organizations National Science Foundation (#1906726), Research Council of Norway (#296836/31406), and National Science Center, Poland (NCN #31382). The work was also partially funded by the Beaufort Lagoon Ecosystems LTER (NSF #1656026 and #2322664) and the Arctic Marine Biodiversity Observing Network (NOAA #NA19NOS0120198, ONF #N00014-22-1-2793, NASA #80NSSC22K1780).]. BB contributed as part of the Kitikmeot Sea Science Study with funding provided by Fisheries and Oceans Canada, Polar Knowledge Canada, and the Arctic Research Foundation.

Please state what role the funders took in the study. If the funders had no role, please state: ''The funders had no role in study design, data collection and analysis, decision to publish, or preparation of the manuscript.''

If this statement is not correct you must amend it as needed.

Please include this amended Role of Funder statement in your cover letter; we will change the online submission form on your behalf.

**The updated financial disclosure statement is:**

**This research was funded through the 2017-2018 Belmont Forum and BiodivERsA joint call for research proposals, under the BiodivScen ERA-Net COFUND program, and with the funding organizations National Science Foundation (#1906726), Research Council of Norway (#296836/31406), and National Science Center, Poland (NCN #31382). The work was also partially funded by the Beaufort Lagoon Ecosystems LTER (NSF #1656026 and #2322664) and the Arctic Marine Biodiversity Observing Network (NOAA #NA19NOS0120198, ONF #N00014-22-1-2793, NASA #80NSSC22K1780). BB contributed as part of the Kitikmeot Sea Science Study (K3S) with funding provided by Fisheries and Oceans Canada, Polar Knowledge Canada, and the Arctic Research Foundation.The funders had no role in study design, data collection and analysis, decision to publish, or preparation of the manuscript.**

4. When completing the data availability statement of the submission form, you indicated that you will make your data available on acceptance. We strongly recommend all authors decide on a data sharing plan before acceptance, as the process can be lengthy and hold up publication timelines. Please note that, though access restrictions are acceptable now, your entire data will need to be made freely accessible if your manuscript is accepted for publication. This policy applies to all data except where public deposition would breach compliance with the protocol approved by your research ethics board. If you are unable to adhere to our open data policy, please kindly revise your statement to explain your reasoning and we will seek the editor's input on an exemption. Please be assured that, once you have provided your new statement, the assessment of your exemption will not hold up the peer review process.

**All data associated with this publication are published and freely available on the Environmental Data Initiative at https://doi.org/10.6073/pasta/48570e835463e405c707a4905c9d172d**

5.Please amend either the title on the online submission form (via Edit Submission) or the title in the manuscript so that they are identical.

**The title on the online submission form was amended.**

6. We note that Figure1 and Supplemental (striking image.tif) in your submission contain [map/satellite] images which may be copyrighted. All PLOS content is published under the Creative Commons Attribution License (CC BY 4.0), which means that the manuscript, images, and Supporting Information files will be freely available online, and any third party is permitted to access, download, copy, distribute, and use these materials in any way, even commercially, with proper attribution. For these reasons, we cannot publish previously copyrighted maps or satellite images created using proprietary data, such as Google software (Google Maps, Street View, and Earth). For more information, see our copyright guidelines: <http://journals.plos.org/plosone/s/licenses-and-copyright>.

We require you to either (1) present written permission from the copyright holder to publish these figures specifically under the CC BY 4.0 license, or (2) remove the figures from your submission:

1. You may seek permission from the original copyright holder of Figure1 and Supplemental (striking image.tif) to publish the content specifically under the CC BY 4.0 license.

We recommend that you contact the original copyright holder with the Content Permission Form (<http://journals.plos.org/plosone/s/file?id=7c09/content-permission-form.pdf>) and the following text:

“I request permission for the open-access journal PLOS ONE to publish XXX under the Creative Commons Attribution License (CCAL) CC BY 4.0 (<http://creativecommons.org/licenses/by/4.0/>). Please be aware that this license allows unrestricted use and distribution, even commercially, by third parties. Please reply and provide explicit written permission to publish XXX under a CC BY license and complete the attached form.”

Please upload the completed Content Permission Form or other proof of granted permissions as an ''Other'' file with your submission.

In the figure caption of the copyrighted figure, please include the following text: “Reprinted from [ref] under a CC BY license, with permission from [name of publisher], original copyright [original copyright year].”

2. If you are unable to obtain permission from the original copyright holder to publish these figures under the CC BY 4.0 license or if the copyright holder’s requirements are incompatible with the CC BY 4.0 license, please either i) remove the figure or ii) supply a replacement figure that complies with the CC BY 4.0 license. Please check copyright information on all replacement figures and update the figure caption with source information. If applicable, please specify in the figure caption text when a figure is similar but not identical to the original image and is therefore for illustrative purposes only.

**The basemap in Fig. 1 was created using the R package ‘grfxtools’, which utilizes Natural Earth (public domain) to project map data. The map was further modified by the authors to show study sites. Thus, the map is within the public domain, is not copyrighted, and complies with the license of PLOS ONE. For more details, see https://github.com/EarthSystemDiagnostics/grfxtools/blob/main/LICENSE.note.**

**5. Review Comments to the Author**

Please use the space provided to explain your answers to the questions above. You may also include additional comments for the author, including concerns about dual publication, research ethics, or publication ethics. (Please upload your review as an attachment if it exceeds 20,000 characters)

**Reviewer #1:**

This manuscript focuses on a highly relevant and ecologically important question: how trophic niche structures in pan-Arctic coastal ecosystems have changed under the dual influence of environmental change and coastal geomorphology. The topic fits well with current global research on Arctic carbon cycling and land–ocean interactions and has clear scientific value. The study compiles and analyses more than 10,000 stable isotope records across multiple trophic levels over the past two decades, showing its strength in data synthesis and regional pattern identification. A particularly commendable contribution is the development and use of the “coastscape” concept, which classifies Arctic coastal systems based on geomorphology, ecological function, and biogeochemical traits. This framework enables meaningful comparisons of consumer isotopic niches across fjords, lagoons, shelves, and straits. The study is a large undertaking with wide spatial and temporal coverage, and it uses appropriate methods such as non-parametric statistics and Bayesian niche overlap modelling (nicheROVER).
However, although the authors demonstrate solid academic ability in building the dataset and analytical framework, some key ecological processes are not discussed in sufficient depth. Generally, the paper does not fully explore the selective assimilation of organic matter by consumers, the role of remineralisation in explaining δ¹⁵N differences in sPOM, or the full pathway of isotopic signal transfer from source to sediment. Also, the assumed δ¹³C values for certain end-members, such as phytoplankton, are somewhat conservative and may lead to misjudging their contribution to consumers. The overall writing is generally clear, but some mechanism discussions lack depth in terms of biogeochemical processes. In summary, I recommend minor revision, as the conclusions are mostly supported by the data and the manuscript is suitable for publication in PLOS ONE after revisions.

**We thank this reviewer for the attention to detail required to provide this constructive feedback that ultimately improved this manuscript. We address these concerns below.**

Specific comments:
Line 48: Please shorten the abstract slightly. Focus on the main message and avoid separating the conclusions.

**The abstract was shortened from 295 words to 255 by distilling the conclusions into overarching ideas.**

Lines 75–77: The phrase “situated in the crucible of climate change” is too rhetorical. Replace it with a more neutral and scientific expression. Similar long or redundant sentences appear elsewhere and should be trimmed for clarity.

**This phrase was removed and the sentence was edited to “This expanse of coastal ecosystems lining the Arctic Ocean are subjected to the impacts of warming-induced changes from both adjacent terrestrial and marine ecosystems.” Now L72.**

Lines 143–146: This is a key sentence. Please highlight the novel aspects of this study, especially regarding trophic niche expression and ecological plasticity.

**To highlight the novelty of the study we added the sentence “Synthesizing this information at the pan-Arctic level is a novel approach to investigate trophic niche variation and explore the ecological plasticity of consumers at the land-sea-ice interface of the coastal Arctic where multiple environmental changes are occurring in tandem.” Now L143-146.**

Line 210: The description of iPOM should be moved to here from Line 213 for better logical flow.

**Done.**

Lines 308–311: Please explain more clearly how “overlap probability” is calculated. A short mathematical description and interpretation would be helpful.

**The text now reads: “As mathematically described by Swanson et al. [30], the method uses a Bayesian framework to estimate the n-dimensional space (N_R_) that a data point has an α probability of being found, where n is the number of isotopes used and α is the user-defined probability, in this case 95%. Then probabilistic niche overlap between two groups is conducted by estimating the probability that a data point randomly selected from one group would fall within the N_R_ space of the other group using a Monte Carlo technique. N_R_ was informed from bivariate δ^13^C and δ^15^N values. The method is robust to differences in sample size, performs well with large sample sizes, and, unlike geometric methods, accounts for species-specific distributions in bivariate space when estimating the probability of overlap [30]. Uncertainty was accounted for by using a Bayesian approach with a noninformative normal-inverse-Wishart prior distribution and 10,000 random permutations to calculate the posterior mean and 95% credible intervals of probabilities of niche overlap.” L305-316.**

Line 359: Although regression results for δ¹³C and δ¹⁵N are reported as significant, model fit indicators (e.g. R² or confidence intervals for Kendall’s tau) should be added in Fig. 2 and the text to strengthen the interpretation.

**On L352-357 the text has been updated to “The linear regressions between collection date and pPOM δ^13^C values (p<0.001, Kendall's τ = -0.267) and collection date and sPOM δ^13^C values (p<0.001, Kendall’s τ = -0.446) revealed significant negative trends with slopes indicating decadal decreases of -2.1 and -2.2 ‰, respectively (Fig 3; Table 4). Likewise, the trend between collection date and sPOM δ^15^N values (p<0.001, Kendall’s τ = -0.478) was significantly negative with a decadal change of -3.1 ‰ per decade (Table 4).”**
**The caption for Fig 3 was also updated accordingly.**

Lines 490–493: Consider adding a rough estimate of freshwater aquatic microalgae input. Based on radiocarbon data, most POM from rivers may be degraded before reaching the coastal ocean (e.g. Goñi et al., 2013).

**Good point. Using the same radiocarbon approach described by Goñi et al. (2013), Behnke et al. (2023) estimated 39-60% of exported river organic matter is derived from aquatic algae. This clarification was added to the text on L499.**

**The text now reads: “Freshwater aquatic microalgae, with δ^13^C values of -33.1±4.7‰, constitute 39-60% of the POM exported by rivers to coastal environments [18].”**

Lines 504–507: The explanation provided is questionable. In the Bering Strait and Chukchi Shelf, terrestrial OM is often more likely to remain suspended in surface waters than marine OM (e.g. Ye et al., 2024). The general statement that terrestrial OM preferentially enters sediments should be reconsidered in light of regional variability. Also, the δ¹⁵N difference between sPOM and pPOM should not be attributed solely to end-member differences. Please consider the influence of mineralisation in surface sediments, including nitrogen regeneration and microbial reworking, and explain why δ¹⁵N is more affected than δ¹³C.

**Good point. Given the results of Ye et al. (2024), we can add this caveat to the mechanisms of OM entering sediments. This paragraph was substantially revised to include caveats and further speculation on the mechanism causing the d15N difference between sPOM and pPOM as mentioned in this comment and the following one. We considered regeneration and remineralization, but we suspect this would cause 15N-enrichment in the sPOM and not 15N-depletion (Montoya 2008). Regardless, further elaboration is given to this surprising finding.**

**Montoya, J. (2008). Nitrogen Stable Isotopes in Marine Environments. In *Nitrogen in the Marine Environment* (2nd ed., pp. 1277–1302). Academic Press.** [**https://doi.org/10.1016/B978-0-12-372522-6.00029-3**](https://doi.org/10.1016/B978-0-12-372522-6.00029-3)

**The new paragraph (L507-531) was updated to:**

**The similar temporal trends of δ13C exhibited by the pPOM and sPOM pools appear to support the tight coupling between the two pools often observed in the Arctic [64,65,82–86]. Therefore, it was unexpected that the δ15N values for the two pools would exhibit different temporal trends (Fig 3). One explanation for no temporal trend for δ15N in pPOM might stem from the timeframe over which it forms and the inorganic nitrogen sources it assimilates compared to the sPOM pool. As the living microalgal component of pPOM blooms during the short Arctic growing season, it would assimilate and reflect the δ15N signature of its inorganic nitrogen pool, typically marine nitrate. Coastal Arctic marine nitrate δ15N values typically range between 5-8‰ [87], and the mean and median values from pPOM in this dataset fall within that range at 6.1 and 6.0‰, respectively. Therefore, we may be observing predominant usage of marine nitrate by coastal primary producers in the pPOM pool over the past two decades, despite recent findings that terrestrial nitrogen fuels about one-third of Arctic primary production [17]. Conversely, sPOM exhibited the trend of 15N-depletion over time, suggesting over the two decades analyzed here its association with marine nitrate weakened while its association with terrestrial nitrogen sources strengthened. Alternatively, the sPOM δ15N trend might not be related to microalgal assimilation of nitrate, but rather a larger proportion of 15N-depleted allochthonous terrestrial OM entering the sPOM pool that is not retained in pPOM. This seems plausible since coastal sediments are repositories of organic matter [88] and accumulate over long periods of time compared to the water column, which constantly refreshes with new microalgal growth. Interestingly, terrestrial OM can remain suspended compared to marine particles that sink faster and contribute more to sPOM over millennial timescales [89], so terrestrial OM deposition near the coast cannot be broadly assumed, especially in deeper zones. Still, biomarker evidence demonstrates that high proportions of sPOM in the coastal Arctic are derived from terrestrial sources [90,91]. Furthermore, it has been noted in lagoons [15], shelves [32,48,58], and fjords [45] that sPOM was consistently 0.4 to 4.0‰ more 15N-depleted than pPOM.**

Lines 513–522: The manuscript connects isotope values of end-members and consumers over time, but does not clearly describe a full mechanism for signal transfer from pPOM → consumer → sPOM. Please outline the steps of fractionation, assimilation, unassimilated residue, and sedimentation more clearly to strengthen the logic.

**We appreciate this comment and the call for additional details. Details and citations were added to the paragraph described in the previous comment regarding the mechanism that connects pPOM to sPOM (L507-531). Briefly, we assume pelagic-benthic coupling is predominant in the shallow, coastal Arctic areas (Dunton et al. 2005; Søreide et al. 2013; McTigue et al. 2015; Stasko et al. 2018; Niemi et al. 2024; Cautain et al. 2024); thus pPOM→sPOM. Undoubtedly, some grazing occurs in the water column with subsequent fecal pellet production, but pPOM remains largely ungrazed until reaching the benthos (Grebmeier et al. 2006 and citations within). Therefore, sPOM is a mixture of sunken pPOM and grazed/reworked pPOM/fecal pellets. However, the grazing/reworking activity would likely cause 15N-enrichment in sPOM, which is not a trend we observe.**

**Dunton, K. H., Goodall, J. L., Schonberg, S. V., Grebmeier, J. M., & Maidment, D. R. (2005). Multi-decadal synthesis of benthic–pelagic coupling in the western arctic: Role of cross-shelf advective processes. *Deep Sea Research Part II: Topical Studies in Oceanography*, *52*(24–26), 3462–3477.** [**https://doi.org/10.1016/j.dsr2.2005.09.007**](https://doi.org/10.1016/j.dsr2.2005.09.007)

**Søreide, J. E., Carroll, M. L., Hop, H., Ambrose, W. G., Hegseth, E. N., & Falk-Petersen, S. (2013). Sympagic-pelagic-benthic coupling in Arctic and Atlantic waters around Svalbard revealed by stable isotopic and fatty acid tracers. *Marine Biology Research*, *9*(9), 831–850.** [**https://doi.org/10.1080/17451000.2013.775457**](https://doi.org/10.1080/17451000.2013.775457)

**McTigue, N. D., Bucolo, P., Liu, Z., & Dunton, K. H. (2015). Pelagic-benthic coupling, food webs, and organic matter degradation in the Chukchi Sea: Insights from sedimentary pigments and stable carbon isotopes: Chukchi Sea sedimentary pigments. *Limnology and Oceanography*, *60*(2), 429–445.** [**https://doi.org/10.1002/lno.10038**](https://doi.org/10.1002/lno.10038)

**Stasko, A. D., Bluhm, B. A., Michel, C., Archambault, P., Majewski, A., Reist, J. D., et al. (2018). Benthic-pelagic trophic coupling in an Arctic marine food web along vertical water mass and organic matter gradients. *Marine Ecology Progress Series*, *594*, 1–19.** [**https://doi.org/10.3354/meps12582**](https://doi.org/10.3354/meps12582)

**Niemi, A., Bluhm, B. A., Juul-Pedersen, T., Kohlbach, D., Reigstad, M., Søgaard, D. H., & Amiraux, R. (2024). Ice algae contributions to the benthos during a time of sea ice change: a review of supply, coupling, and fate. *Frontiers in Environmental Science*, *12*, 1432761.** [**https://doi.org/10.3389/fenvs.2024.1432761**](https://doi.org/10.3389/fenvs.2024.1432761)

**Cautain, I. J., Last, K. S., Bluhm, B. A., Renaud, P. E., McKee, D., & Narayanaswamy, B. E. (2024). High uptake of sympagic organic matter by benthos on an Arctic outflow shelf. *PLOS ONE*, *19*(8), e0308562.** [**https://doi.org/10.1371/journal.pone.0308562**](https://doi.org/10.1371/journal.pone.0308562)

**Grebmeier, J. M., Cooper, L. W., Feder, H. M., & Sirenko, B. I. (2006). Ecosystem dynamics of the Pacific-influenced Northern Bering and Chukchi Seas in the Amerasian Arctic. *Progress in Oceanography*, *71*(2–4), 331–361.** [**https://doi.org/10.1016/j.pocean.2006.10.001**](https://doi.org/10.1016/j.pocean.2006.10.001)

Line 598: Please recheck the δ¹³C range for Arctic coastal phytoplankton (−26‰ to −23‰), as this may be too low. Several studies report values of about −21‰ or higher in shallow productive waters (e.g. Søreide et al., 2006), and underestimating this range may lead to underestimating phytoplankton contributions.

**Thank you for pointing this out to us. We completely agree with this comment. Indeed Arctic coastal phytoplankton can be as heavy as -20 ppt, as we show in Fig 2 and Table 3. The issue was that the text was unclear; it has now been modified for clarity. L616-623**

“Phytoplankton are a common constituent of the pPOM mixture in most marine habitats and may sink and become incorporated into the sPOM pool [Søreide et al. 2013, Stasko et al. 2018, McTigue et al. 2015]. Within the pPOM aggregation in this dataset, data rows specifically called phytoplankton ranged from -26.7‰ to -20.3‰ for δ13C. This matches other reported values for coastal Arctic phytoplankton of -26‰ to -19‰ [McMahon et al. 2006, McMahon et al. 2021, Yu et al. 2023]. However, the range of phytoplankton stable isotope values (-26‰ to -19‰) cannot solely account for the range of pPOM (-33.5‰ to -19.8‰) and sPOM (-28.5‰ to -19.0‰) reported here. Therefore, other 13C-depleted carbon sources relative to phytoplankton must have contributed to the mixture of POM pools.”

**The following reference was added to provide evidence that phytoplankton values reported by our study overlap with the literature:**

Yu, X., Ye, L., Bian, Y., Liu, Y., Zhang, W., Wang, W., et al. (2023). Composition and sources of suspended particles in the Pacific Arctic region. *Marine Environmental Research*, *191*, 106127.<https://doi.org/10.1016/j.marenvres.2023.106127>

Lines 599–601: Please discuss the possibility that consumers preferentially assimilate ¹³C-rich compounds (e.g. proteins), which could cause consumer δ¹³C values to increase while leaving behind ¹³C-depleted lipids in the remaining pPOM.

**We were unable to find a clear mechanism for the possibility of preferential protein assimilation paired with lipid rejection in the literature. There are many instances of the usage of fatty acids (a lipid) for tracing assimilation pathways in Arctic consumers from different algal sources (e.g., Budge et al. 2008; Wang et al. 2014; Mohan et al. 2016). The success of this methodology indicates that the proportion of lipid assimilation from algal sources like pPOM is high, with only a negligible fraction of lipids shunted to the pPOM/sPOM pools through sloppy feeding or fecal pellets. Moreover, the timing and quality of lipid assimilation by consumers is critical for survival of Arctic species (Søreide et al. 2010; Leu et al. 2011) suggesting that preferential rejection of the lipid fraction in Arctic algal sources while grazing is not likely. Despite this, there still remains the possibility that preferential assimilation and/or isotopic routing occurs that cannot be deciphered by bulk stable isotope analysis, which is what this study utilizes. Moreover, the broad feeding modes attributed to consumers and large geographic scope used in this study lends itself to further possibility that species-specific differences in isotopic discrimination, isotopic routing to different tissue types, selective feeding, selective assimilation, consumption without assimilation, and differential assimilation/discrimination of mixed diets could occur without detection (Robins et al. 2009, Doi et al. 2006, Dodds et al. 2014). This comment sheds light on the caveats of the methodology used in this study, which we have added to the text.**

**The new text reads on L686-692:**

“We acknowledge that the use of bulk stable isotopes, broad feeding habit categories, and wide geographic coverage leaves open the possibility that species-specific selective assimilation, isotopic routing between tissue types, and differences in isotopic discrimination could occur without detection by the methods employed by this study[Robins et al. 2009, Doi et al. 2006, Dodds et al. 2014]. However, we argue the comparisons of bulk stable isotope values between consumers and organic matter sources described above are robust to individual or species-level instances of atypical stable isotope behavior due to the volume of aggregated data used to parse broad patterns.”

**References:**

Budge, S. M., Wooller, M. J., Springer, A. M., Iverson, S. J., McRoy, C. P., & Divoky, G. J. (2008). Tracing carbon flow in an arctic marine food web using fatty acid-stable isotope analysis. *Oecologia*, *157*(1), 117–129.<https://doi.org/10.1007/s00442-008-1053-7>

Wang, S. W., Budge, S. M., Gradinger, R. R., Iken, K., & Wooller, M. J. (2014). Fatty acid and stable isotope characteristics of sea ice and pelagic particulate organic matter in the Bering Sea: tools for estimating sea ice algal contribution to Arctic food web production. *Oecologia*, *174*(3), 699–712.<https://doi.org/10.1007/s00442-013-2832-3>

Mohan, S. D., Connelly, T. L., Harris, C. M., Dunton, K. H., & McClelland, J. W. (2016). Seasonal trophic linkages in Arctic marine invertebrates assessed via fatty acids and compound-specific stable isotopes. *Ecosphere*, *7*(8), e01429.<https://doi.org/10.1002/ecs2.1429>

Søreide, J. E., Leu, E., Berge, J., Graeve, M., & Falk-Petersen, S. (2010). Timing of blooms, algal food quality and *Calanus glacialis* reproduction and growth in a changing Arctic: Timing and production in a changing Arctic. *Global Change Biology*, *16*, 3154–3163.<https://doi.org/10.1111/j.1365-2486.2010.02175.x>

Leu, E., Søreide, J. E., Hessen, D. O., Falk-Petersen, S., & Berge, J. (2011). Consequences of changing sea-ice cover for primary and secondary producers in the European Arctic shelf seas: Timing, quantity, and quality. *Progress in Oceanography*, *90*(1–4), 18–32.<https://doi.org/10.1016/j.pocean.2011.02.004>

Robbins, C. T., Felicetti, L. A., & Florin, S. T. (2010). The impact of protein quality on stable nitrogen isotope ratio discrimination and assimilated diet estimation. *Oecologia*, *162*(3), 571–579.<https://doi.org/10.1007/s00442-009-1485-8>

Doi, H., Kikuchi, E., Takagi, S., & Shikano, S. (2006). Selective assimilation by deposit feeders: Experimental evidence using stable isotope ratios. *Basic and Applied Ecology*, *7*(2), 159–166.<https://doi.org/10.1016/j.baae.2005.04.011>

Dodds, W. K., Collins, S. M., Hamilton, S. K., Tank, J. L., Johnson, S., Webster, J. R., et al. (2014). You are not always what we think you eat: selective assimilation across multiple whole-stream isotopic tracer studies. *Ecology*, *95*(10), 2757–2767.<https://doi.org/10.1890/13-2276.1>

**Reviewer #2:**

This study integrates two decades of pan-Arctic coastal ecosystem data to explore spatiotemporal changes in consumer isotope niches under climate change, with significant ecological and environmental implications. Three null hypotheses (stability of isotope values over decades, consistency of isotope niches between coastal landscapes, and regional differences within coastal landscapes) provide a clear analytical framework.
The significant decrease in δ13C values of pPOM/sPOM (2.1‰–2.2‰ per decade) and the parallel trends in consumers strongly support the hypothesis of increased terrestrial organic matter input, providing quantitative evidence for the trend collected in recent years at sampling sites in the high latitudes of the Arctic.Is the “terrestrialization”?
How have different landscapes at similar or close latitudes varied isotopically over the past two decades?

**We thank the reviewer for their comments.**

**In terms of identifying decadal trends in isotopes in different landscapes at similar latitudes:**

**Delving into the terrestrial side of these changes is outside the scope of this study, which focused on the coastal environment. There is strong evidence by Rawlins and Karmalkar (2024) and Stadnyk et al. (2021) that increases in river discharge and subsurface flow in the terrestrial Arctic landscape liberate terrestrial organic matter and deliver it to the coastal ecosystems, which is part of what we observe in this study. While we focused on the marine coastscapes, de la Vega et al. (2019) showed similar isotopic trends in the Arctic pelagic systems; their results are described in the Discussion (see section titled “Temporal changes in stable isotope values”).**

Comments
1. Add keywords;

**PLOS ONE did not request keywords upon manuscript submission. A list will be generated if the Editor requests it.**

2. Label place names in Figure 1;

**Done.**

3. Lines 556 to 561, 574 to 577
The influence of the Alaska Coastal Current on the distribution and transport of organic matter and its potential impact on isotopic signatures in coastal food webs should also be considered.

**We inadvertently overlooked the influence of the Alaska Coastal Current/Alaska Coastal Water, but it should definitely be considered here. It is not included in the text on L579 and L596.**
